# Evidence for Deep Regulatory Similarities in Early Developmental Programs across Highly Diverged Insects

**DOI:** 10.1093/gbe/evu184

**Published:** 2014-08-29

**Authors:** Majid Kazemian, Kushal Suryamohan, Jia-Yu Chen, Yinan Zhang, Md. Abul Hassan Samee, Marc S. Halfon, Saurabh Sinha

**Affiliations:** ^1^Department of Computer Science, University of Illinois at Urbana-Champaign; ^2^Laboratory of Molecular Immunology, National Heart Lung and Blood Institute, National Institutes of Health, Bethesda, Maryland; ^3^Department of Biochemistry, University at Buffalo-State University of New York; ^4^NY State Center of Excellence in Bioinformatics and Life Sciences, Buffalo, New York; ^5^Department of Biological Sciences, University at Buffalo-State University of New York; ^6^Molecular and Cellular Biology Department and Program in Cancer Genetics, Roswell Park Cancer Institute, Buffalo, New York; ^7^Institute of Genomic Biology, University of Illinois at Urbana-Champaign

**Keywords:** cross species enhancer discovery, regulatory modules in diverged insects, alignment free enhancer prediction

## Abstract

Many genes familiar from *Drosophila* development, such as the so-called gap, pair-rule, and segment polarity genes, play important roles in the development of other insects and in many cases appear to be deployed in a similar fashion, despite the fact that *Drosophila*-like “long germband” development is highly derived and confined to a subset of insect families. Whether or not these similarities extend to the regulatory level is unknown. Identification of regulatory regions beyond the well-studied *Drosophila* has been challenging as even within the Diptera (flies, including mosquitoes) regulatory sequences have diverged past the point of recognition by standard alignment methods. Here, we demonstrate that methods we previously developed for computational *cis*-regulatory module (CRM) discovery in *Drosophila* can be used effectively in highly diverged (250–350 Myr) insect species including *Anopheles gambiae*, *Tribolium castaneum*, *Apis mellifera*, and *Nasonia vitripennis*. In *Drosophila*, we have successfully used small sets of known CRMs as “training data” to guide the search for other CRMs with related function. We show here that although species-specific CRM training data do not exist, training sets from *Drosophila* can facilitate CRM discovery in diverged insects. We validate in vivo over a dozen new CRMs, roughly doubling the number of known CRMs in the four non-*Drosophila* species. Given the growing wealth of *Drosophila* CRM annotation, these results suggest that extensive regulatory sequence annotation will be possible in newly sequenced insects without recourse to costly and labor-intensive genome-scale experiments. We develop a new method, Regulus, which computes a probabilistic score of similarity based on binding site composition (despite the absence of nucleotide-level sequence alignment), and demonstrate similarity between functionally related CRMs from orthologous loci. Our work represents an important step toward being able to trace the evolutionary history of gene regulatory networks and defining the mechanisms underlying insect evolution.

## Introduction

**Fig. 1.—:**
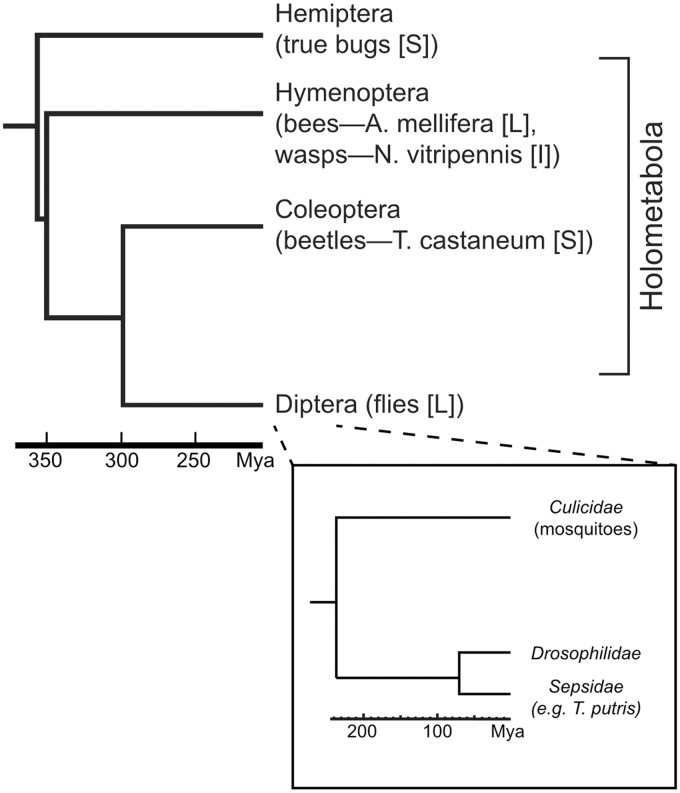
Phylogeny and evolutionary divergence of the Holometabola. For clarity, only species used in this study are shown, along with the closest nonholometabolous insect order, the Hemiptera. Inset shows the Dipteran (fly) radiation. Letters in brackets indicate mode of development for the indicated species: S, short germband; L, long germband; I, intermediate (mix of short and long characteristics). Divergence times are from Wiegmann et al. (2011) for the Diptera and from Wiegmann et al. (2009) for the other orders.

**Fig. 2.—:**
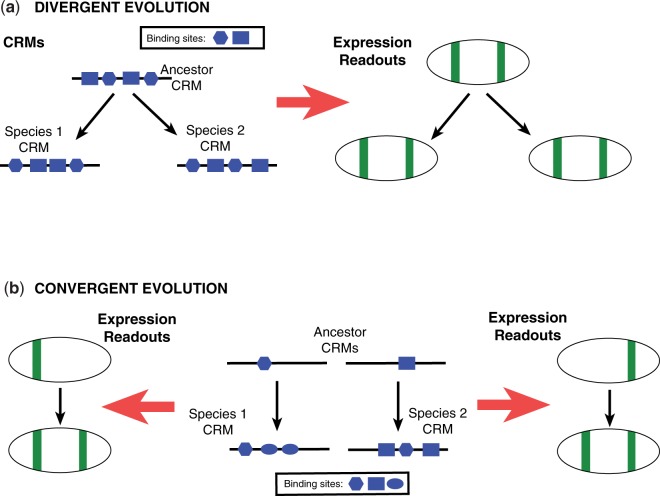
Schematic of enhancer evolution modes. Homologous pair of enhancers derived by direct descent (*a*) from a common ancestral enhancer or by convergent evolution (*b*) from different sequences in the ancestor. Black arrows show evolutionary relationships. Red arrows indicate expression driven by the CRM in an idealized fly embryo, with green indicating activity. Each shape (rectangle, oval, hexagon) indicates binding sites of a different TF. (*b*) CRM in the ancestral genome diverges in terms of the arrangement of binding sites but conservation of site composition ensures that the regulatory output is conserved. (*b*) Two different sequences in the ancestral genome convergently evolve to extant CRMs with different site compositions but similar expression readouts. In either case, the two derived CRMs are unlikely to be alignable at the nucleotide level, in one case (*a*) due to contrasting arrangements of similar binding sites and in the other case (*b*) due to different site compositions. Note: Evolution modes shown here are only two toy examples representing a broad spectrum of possibilities.

The early embryonic development of *Drosophila melanogaster* (*D. mel*; “fruit fly”) has been the subject of intensive study, resulting in a sophisticated, albeit incomplete, understanding of the genes involved in pattern formation and extensive characterization of the transcriptional *cis*-regulatory modules (CRMs) that regulate them. A growing number of studies in divergent insect species, including the Hymenopterans *Apis mellifera* (*A. mel*; honeybee) and *Nasonia vitripennis* (*N. vit*; jewel wasp), and the Coleopteran *Tribolium castaneum* (*T. cas*; red flour beetle), have began to build on this knowledge to explore how developmental gene regulatory networks have evolved over the course of insect diversification. These studies have revealed both striking similarities and significant differences in the mechanisms of early development among these species (Peel 2008; Rosenberg et al. 2009; Lynch et al. 2012). Intriguingly, many of the observed similarities and differences seem to track less well with phylogeny than with mode of embryonic development. Thus *A. mel* and *N. vit*, which as members of the Hymenoptera belong to the most basal clade of the holometabolous insects (fig. 1), appear to be more similar to *Drosophila* in terms of gap and pair-rule gene expression than does the more closely related beetle *T. cas*. It has been suggested (Peel 2008) that this is due to the fact that both *A. mel* and *N. vit* develop, like *Drosophila*, using a long-germband mode of embryogenesis (in essence, with most or all segments having their fates established simultaneously prior to gastrulation), whereas *T. cas* undergoes short-germband development, with progressive determination of segmental fates. This has raised the possibility that the long-germband mode, which is not found outside of the Holometabola, evolved independently in the hymenopteran and dipteran lineages (Peel 2008). In this case, similarities in gap and pair-rule gene expression and regulation might represent convergent evolution, with regulatory relationships and CRMs of comparable function arising de novo and perhaps aided by co-option of transcription factors (TFs) from a different ancestral regulatory program (Heffer and Pick 2013) (fig. 2*b*). Alternatively, developmental gene expression and regulation in each of these insects could derive by direct descent from the ancestral genome (fig. 2*a*), with turnover of TF binding sites in orthologous CRM sequences and changes in transacting TFs in the individual lineages leading to the observed differences in their developmental genetics (Lynch, Brent, et al. 2006; Williams et al. 2008).

A key component to understanding regulatory network evolution is analysis of the CRMs that integrate the activities of the orthologous developmental genes. For instance, establishing that orthologous CRMs regulate orthologous genes at multiple steps during development would support the direct descent model, whereas showing use of newly acquired CRMs, or CRMs with distinct binding site compositions and TF combinations, would favor the convergent evolution model. The limited examples of developmental CRMs available today, scattered across different studies, generally point to a scenario of ancestrally derived regulation (Erives and Levine 2004; Zinzen et al. 2006; Goltsev et al. 2007; Cande, Chopra, et al. 2009; Cande, Goltsev, et al. 2009). However, they do not allow us to systematically determine the extent of regulatory network conservation at the level of *cis*-regulatory mechanism.

Unfortunately, identifying related—by either convergent or direct evolution—CRMs for such a systematic study has presented a considerable challenge. Even among species of the same order, noncoding sequences have diverged beyond the point where standard alignment algorithms are effective, making it impossible to identify homologous noncoding sequences through sequence comparison alone. Computational methods based on clustered TF binding sites (motifs) have proven effective in a small number of cases, primarily from studies of dorsal–ventral patterning (Erives and Levine 2004; Zinzen et al. 2006; Goltsev et al. 2007; Cande, Goltsev, et al. 2009). However, in general such methods have a high false-positive rate, and sufficient knowledge of TFs and motifs is rarely present outside of a few well-studied biological processes (Haeussler and Joly 2011).

Our previous work (Kantorovitz et al. 2009; Kazemian et al. 2011) offers a practical solution to this CRM discovery problem: it does not rely on TFs or their binding motifs, but instead exploits the observation that for many regulatory systems of interest, a small set of known CRMs can be used as training data to facilitate a more complete characterization of related CRMs. We have previously shown (Kantorovitz et al. 2009; Kazemian et al. 2011) that when “training CRMs” from a particular regulatory network are available, this approach is superior to or as effective as existing motif-based methods (Frith et al. 2003) and other methods that use alignment-free scores of sequence similarity (Arunachalam et al. 2010). However, the requirement for training data represents a serious shortcoming. Although extensive data for over 1,850 CRMs, complete with sequences and associated gene expression patterns, are available for *D. mel* (Gallo et al. 2011), such information is less easily accessible for other model organisms and virtually nonexistent for emerging-model and nonmodel species. For instance, our search of the literature revealed fewer than 20 well-defined CRMs collectively for the three species *An. gam*, *T. cas*, and *A. mel*, and none for *N. vit* (see supplementary note S1, Supplementary Material online). We wondered whether this problem could be overcome by using the wealth of available *Drosophila* data to conduct cross-species CRM discovery in other, diverged insect species. That this would be feasible was by no means certain a priori: the assumptions we had previously tested about sequence similarity of functionally similar CRMs within *Drosophila* would now be put to the test across species separated by up to 350 Myr of insect evolution. (It is worth noting, moreover, that insects, perhaps due to their shorter generation times, have been shown to have a faster rate of genome evolution than vertebrates. For example, flies and honeybees, with their 350-Myr divergence, have only ∼10% of orthologous genes in synteny compared with the 50% seen with the 450 Myr human–pufferfish divergence [Zdobnov and Bork 2007].)

We show here that *Drosophila* training data indeed can enable effective CRM discovery in these species despite their great divergence. We predict over 7,100 new CRMs in four non-Drosophilid insect species and validate 12 of 16 of these as functional in vivo by transgenic reporter gene assays in *Drosophila*. Many of these drive gene expression in a pattern strikingly similar to a known *Drosophila* CRM from the orthologous locus, strongly suggesting that we successfully identified functionally related CRMs despite sequence divergence to the point where the sequences cannot be aligned. Further support for this conclusion comes from analysis of the motif composition of the newly discovered CRMs, which show substantial similarity in motif content and in some cases, motif organization, when compared with CRMs in the orthologous loci in *Drosophila* (again despite the absence of alignable blocks of conserved sequence). Thus, we find that similar gene expression in highly diverged species is often regulated through similarly constituted CRMs. Our work also suggests that regulatory sequence annotation of diverse insects is an attainable goal without requiring extensive new genome-scale experimental data for each newly sequenced species, and provides a valuable starting point for future attempts to reconstruct the evolutionary history of developmental regulatory networks spanning over 350 Myr of evolution.

## Materials and Methods

### Statistical Scores

Each statistical model presented below scores a target sequence window *S* for similarity, in terms of k-mer composition, to a training set of known CRMs from *Drosophila* (see supplementary file S1, Supplementary Material online), denoted by *C*. These scores are described in detail in Kantorovitz et al. (2009) and Kazemian et al. (2011), and we only outline their main features here. The source codes of the methods are provided in supplementary file S2, Supplementary Material online.

#### msHexMCD

A fifth-order Markov chain is trained on the set of sequences in the training set *C* as well as their orthologs from 11 other *Drosophila* genomes. In particular, let *N_w_* denote the count of 6-mer *w* in sequences in *C* and their orthologs. This count is first smoothed based on counts *N_w__′_* of every 6-mer *w′* that is one mismatch away from *w*. Thus, the smoothed count of *w* is given by ${\tilde{N}}_{w}=N_{w}+0.25\times \sum_{w^{'}}N_{w^{'}}$. Smoothed counts ${\tilde{N}}_{w}$ of all 6-mers *w* are used to train a fifth-order Markov chain $M^{+}$. A different fifth-order Markov chain $M^{-}$ is trained on a set of background sequences, also taken from *Drosophila*. The score of sequence *S* is the log-likelihood ratio (LLR) of the two Markov chains on *S*, that is, msHexMCD(*S*) = log(Pr[*S*|$M^{+}$]/Pr[*S*|$M^{-}$]).

#### msIMM

We trained a fifth-order interpolated Markov model (IMM^+^) (introduced by Salzberg et al. [1998] and re-implemented in Kazemian et al. [2011]) on the set of sequences in the training set *C* as well as their orthologs from 11 other *Drosophila* genomes. An IMM combines all Markov chains of up to certain order (here 5). It provides a natural extension to a fixed order Markov chain and can better capture the natural variability of TF binding sites (see Kazemian et al. 2011 for more details). An IMM (IMM^−^) is trained separately on a set of background sequences. The score of sequence *S* is the LLR of the two IMMs on *S*, that is, msIMM(S) = log(Pr[*S*|IMM^+^]/Pr[*S*|IMM^−^]).

#### PAC-rc

This score quantifies the overrepresentation of every 6-mer w∈W in sequences in *C*, relative to a set of background sequences, assuming a Poisson distribution of word counts. The score is based on van Helden (2004) and reimplemented in Kantorovitz et al. (2009). Let $n_{w}\text{ and }\lambda_{w}$ denote the count of *w* in *C* and a size-matched set of background sequences, respectively. The score of sequence *S* is defined as: PAC-rc(*S*) = $\frac{1}{\left|W\right|}\sum_{w\in W}F\left(\lambda_{w},n_{w}-1\right)$, where F(λ,.) is the Poisson cumulative distribution function (CDF) with mean λ.

### Global Assessment Using Expression Gene Sets: The “Evaluation *P* Value”

As an initial assessment, we statistically examined whether the predicted CRMs of a regulatory system in a non-*Drosophila* (target) genome are located near the genes related to that system. Here, a regulatory system refers to an expression domain or stage, the genes expressed in that domain or stage (expression gene set), and the CRMs that drive expression in the domain or stage. For each regulatory system and each non-*Drosophila* target genome, we first inferred the expression gene sets *G* from relevant expression gene sets in *Drosophila* using homology maps downloaded from the Inparanoid database (Ostlund et al. 2010). For each target genome, we also created a gene universe *U* as all genes with a homolog in *Drosophila* according to the Inparanoid database. The expression gene sets and universes are provided in supplementary file S1, Supplementary Material online. Given a collection of predicted CRMs, we collected the nearest neighboring gene for each of the top scoring windows until this set of genes, *P*, is of size 200 genes. We finally examined the statistical significance using a Hypergeometric test H(*n*, *P*, *G*, *U*), where *n* is the number of genes in both *P* and *G*. We call this the evaluation *P* value. (We used a fixed number of genes in set *P* rather than genes defined by a score threshold, in order to ensure that *P* values from different Hypergeometric tests are based on the same sample size. The size of 200 genes was chosen following Kantorovitz et al. [2009].)

### *Drosophila* Reporter Constructs and Transgenic Analysis

Genomic sequences were generated by polymerase chain reaction using genomic DNA from the appropriate species, cloned into pJet1.2blunt (Fermentas), and confirmed by sequencing. Primer sequences are provided in supplementary table S8, Supplementary Material online. The putative CRM sequences were subcloned into plasmid pattBnucGFPs, a ϕC31-enabled *Drosophila* transformation vector containing Enhanced Green Fluorescent Protein (EGFP) under the control of a minimal *hsp70* promoter (details available on request), using *Xba*I + *Xho*I. Transgenic flies were produced by Genetic Services Inc. (Cambridge, MA) by injection into lines attP2 or attP40. Homozygous transgenic embryos were collected, fixed, and stained using standard methods.

### Binding Site Annotation and Quantitative Modeling for *hairy* and *sog* CRMs

A state-of-the-art sequence-to-expression model, GEMSTAT (He et al. 2010), was used to predict the expression readouts of the *hairy* and *sog* CRMs. GEMSTAT uses motifs and relative concentration profiles of TFs to model CRM-driven expression patterns. The model can fit data under various mechanistic assumptions, for example, whether transcriptional repressors work over long or short distances, whether cooperativity (direct or indirect, homotypic or heterotypic) exists between CRM-bound TF molecules, etc. To annotate binding sites for a TF in a given CRM, we first compute the LLR score of each *k*-bp window in the CRM, where *k* denotes the TF’s motif length and the two likelihoods are computed from the TF’s PWM (position weight matrix) and a uniform background distribution. We then annotate binding sites as those with LLR score greater than 0.5 times the LLR score of the optimal site (maxLLR score) of the corresponding TF. In our experience of working with other data sets of *D. mel* developmental gene regulation, the threshold 0.5 helped us capture most of those weak sites that are not too degenerate and hence may be assumed to bear functional importance. We have visualized the annotated binding sites using the inSite tool (Miriah) that depicts each site as a rectangle with the base and the height representing the length and the strength (computed as ten times the LLR/maxLLR ratio) of the site, respectively. For CRM alignments, CRM sequences and/or those of orthologous regions were extended up to 1 kb to each side of the CRM prior to binding site annotation.

The hairy CRM readout was predicted from the GEMSTAT model reported in He et al. (2010), where it was trained on 37 enhancers involved in the early patterning of the anterior–posterior axis in the *D. mel* embryo. The input TFs to the model were BICOID, CAUDAL, GIANT, KNIRPS, HUNCHBACK, and KRUPPEL. The motifs used in this and the following model were all collected from FlyFactorSurvey (Zhu et al. 2011), except the BICOID motif, which was taken from FlyREG (Bergman et al. 2005) and the ZELDA motif, where we changed the FlyFactorSurvey PWM to match the motif presented in Nien et al. (2011). Expression profiles and TF concentration profiles used in training and prediction were 60-dimensional vectors of relative values on a scale of 0–1, with dimensions corresponding to evenly placed positions along the A/P axis from 20% to 80% egg length. The model assumed homotypic cooperativity for BICOID and KNIRPS, and that repressors act over long distances to regulate transcription of their target genes. The model used to predict the *sog* CRM readout was trained on the *Drosophila ind* and *sog* (shadow) enhancers (Stathopoulos and Levine 2005; Hong et al. 2008). TFs input to the model were DORSAL, SNAIL, and ZELDA. The model assumed heterotypic cooperativity between DORSAL and ZELDA, and that repressors can regulate transcription by acting over long distances.

### Regulus

(Complete details in supplementary file S3, Supplementary Material online.) Here, we compute a probabilistic score of similarity between two sequences *x* and *y* in terms of their binding site composition. This score allows for the uncertainty of binding site prediction and for the varying relevance of different types of binding sites. It is a likelihood ratio of a “homology model” denoted by “*M*,” and a null model denoted by “*nB.*” In the null model, the sequences *x* and *y* are independently generated by a zeroth-order hidden Markov model (HMM) similar to Sinha et al. (2003). The HMM has one state for each input motif *m* and one background state *bg*. The HMM stochastically enters a state, stochastically emits a single nucleotide (if the state is *bg*) or a single site (if in a motif state), and repeats this process until it has emitted a nucleotide sequence of the predetermined length, that is, length of *x* or *y*. Transition probabilities for each state are independent of the current state and depend only on the state being entered. These transition probabilities are learned by maximum-likelihood estimation from an appropriate set of training CRMs and reflect the density of motif matches in those CRMs. After estimation, we adjust the motif transition probabilities by scaling by a constant so that the average of all motif transition probabilities is 0.001. (Details at the end of this section.) Emission probabilities in the background state are determined by nucleotide frequencies in the given sequences, and those in a motif state are determined by the PWM form of the motif. Let *T* represents the sequence of (hidden) states entered by the HMM in generating the two sequences *S* = *{x*, *y}*, then Pr(*T|HMM)* denotes the product of all transition probabilities corresponding to *T*, and Pr(*S|T*) is the probability of emitting the given sequences conditional on *T*. The likelihood of *S* under the null model *nB* is then given by

$$\mathrm{Pr}\left(S|nB\right)=\underset{T}{\sum }\mathrm{Pr}\left(T|HMM\right)\text{Pr}(S|T)$$

with the sum being taken over all possibilities of the hidden state path *T* generating both sequences.

The homology model is very similar to the above, with the exception that the probability of a state path *T*, that is, Pr(*T*) above, is now scaled by a multiplicative factor φ(T) that reflects the similarity of motif composition of the two sequences, according to *T*. In particular, if *T* includes $n_{m}^{x}$ visits to the motif state *m* in generating sequence *x* and $n_{m}^{y}$ visits to the same motif state in generating sequence *y*, that is, if *T* prescribes $n_{m}^{x}$ sites of *m* in *x* and $n_{m}^{y}$ sites of *m* in *y*, then

$$\varphi \left(T\right)=\varphi^{\underset{m}{\sum }\text{min}(n_{m}^{x},n_{m}^{y})},$$

where φ is a constant (set to 5 by default). Thus, the similarity score induced by the stochastic (and hidden) state path *T* is an exponential function of the shared numbers of sites of each motif, summed over all motifs. Now, the probability of state path *T* under the homology model s defined as being proportional to φ(T)Pr(T|HMM), that is

$$\mathrm{Pr}\left(T\text{|}M\right)=\frac{\text{φ}(\text{T})\text{Pr}(T|HMM)}{\sum_{T^{'}}\varphi \left(T^{'}\right)\text{Pr}(T^{'}|HMM)}.$$

Finally, the likelihood of *S* under the homology model *M* is given by

$$\mathrm{Pr}\left(S\text{|}M\right)=\underset{T}{\sum }\mathrm{Pr}\left(T\text{|}M\right)\text{Pr}(S|T).$$

The Regulus score for the given pair of sequence *S* = {*x*,*y*} is the LLR given as follows:

$$\text{LLR}=\log\frac{\text{Pr}(S|M)}{\text{Pr}(S|nB)}.$$

Everything else being equal if one pair of sequences has more shared sites for a motif *m*, that is, a greater value of $\text{min}(n_{m}^{x},n_{m}^{y})$, the LLR score will be greater (supplementary file S3, Supplementary Material online). Note that the null model *nB* assumes the same HMM, rather than two separate HMMs, have generated the pair of sequences. This corresponds to the null hypothesis that the two sequences independently mirror some aspects of the binding site composition of training CRMs, and thus provides a strong baseline to contrast with the homology model where they share sites for the same motifs.

See supplementary file S3, Supplementary Material online, for details of Regulus. (Note that the LLR score above is referred to as LLRB, and state paths *T* are denoted by the lower case *t* in the supplementary file S3, Supplementary Material online.)

#### Motifs Used in Regulus

For A/P patterning CRMs we used motifs for the TFs BCD, CAD, GT, HB, KNI, KR, HKB, TLL, FKH, and CIC and for D/V patterning CRMs we used motifs for TFs DL, BRK, SNA, VND, IND, CIC, TWI, ZEN, and ESPL. All motifs were obtained from FlyFactorSurvey (Zhu et al. 2011).

#### Transition Probabilities in Regulus

For each motif, a motif density was estimated by training a two-state HMM (one state representing motif and one state for background), using the Baum–Welch algorithm, on an appropriate set of sequences. We used the training set of CRMs associated with A/P patterning and D/V patterning (“ap” and “dv,” respectively, supplementary file S1, Supplementary Material online) in this step. The motif densities were then scaled so as to have an average of 0.001, to obtain transition probabilities in the combined HMM (with one state for each motif) mentioned above. Thus, if there are *k* motifs, the sum of motif transition probabilities in the final HMM is *k* × 0.001, and the remaining probability mass is assigned to the background state.

### BLAST Analysis and Dot Plot Alignments

Intergenic sequences flanking each developmental gene were extended up to 20 kb, terminating at the beginning of the next coding region. Sequence pairs of *D. mel* plus another species were then analyzed for sequence similarity using the NCBI (National Center for Biotechnology Information) BLAST server (Boratyn et al. 2013) with parameters customized for improved cross-species comparison (-W 9, -G 1, -E 2, -r 1, -q -1; see Korf et al. 2003). For each *D. mel* sequence, BLAST was performed 1) against the orthologous *D. mojavensis* or *D. virilis* locus (for *twi*, *D. willistoni*); 2) against the orthologous locus from one or more of *An. gam*, *T. cas*, *N. vit* and *A. mel*; and 3) against 1,000 randomized sequences obtained by shuffling the respective non-*D. mel* sequence. For *even skipped* alignments, sequence from *Themira putris* (Hare et al. 2008) was also analyzed. To gauge the distribution of the bit scores of the resulting BLAST High Scoring Pairs (HSPs), box plots (showing median, inter-quartile range (IQR), 1.5 × IQR) of the top 50 BLAST hits for each of the BLAST runs were generated and statistical significance was tested by performing pairwise comparisons using Tukey’s multiple comparison test (see supplementary table S9, Supplementary Material online, for contingency matrix of *P* values).

Dot plot alignments were generated using the EMBOSS *dottup* program (Rice et al. 2000) with window size = 9 and step size = 1.

## Results

### Many Developmental Genes Show Conservation in Expression but No Simple Conservation in Regulatory Sequences

As a first step toward identifying potentially homologous CRMs, we assessed similarity in developmental gene expression among *A. mel*, *N. vit*, *T. cas*, and *An. gam*, drawing on reports of over 75 different genes whose expression has been examined in one or more of these species in addition to *Drosophila* (supplementary table S1, Supplementary Material online). Although there exist some notable changes in expression patterns (e.g., in the genes of the terminal patterning system [Duncan et al. 2013], or in the more ventralized expression of *sog* in mosquitoes vs. flies discussed below), the vast majority of the genes analyzed showed overall similarity in expression to their *Drosophila* orthologs during at least some stages of embryogenesis. This includes gap and pair-rule genes (Bucher and Klingler 2004; Eckert et al. 2004; Pultz et al. 2005; Lynch, Brent, et al. 2006; Lynch, Olesnicky, et al. 2006; Olesnicky et al. 2006; Wratten et al. 2006; Aranda et al. 2008; Cerny et al. 2008; Nunes da Fonseca et al. 2008; Schetelig et al. 2008; Schinko et al. 2008; Choe and Brown 2009; Keller et al. 2010; Lynch and Desplan 2010; Wilson et al. 2010; Wilson and Dearden 2011, 2012; Duncan et al. 2013), the segment polarity genes *wg* and *engrailed* (Nie et al. 2001; Lynch, Brent, et al. 2006; Choe and Brown 2009; Duncan et al. 2013), *Sox* family members (Wilson and Dearden 2008), and others (Papatsenko et al. 2011). Thus, it is reasonable to hypothesize that regulatory relationships and CRM sequences may also be similar.

**Fig. 3.—:**
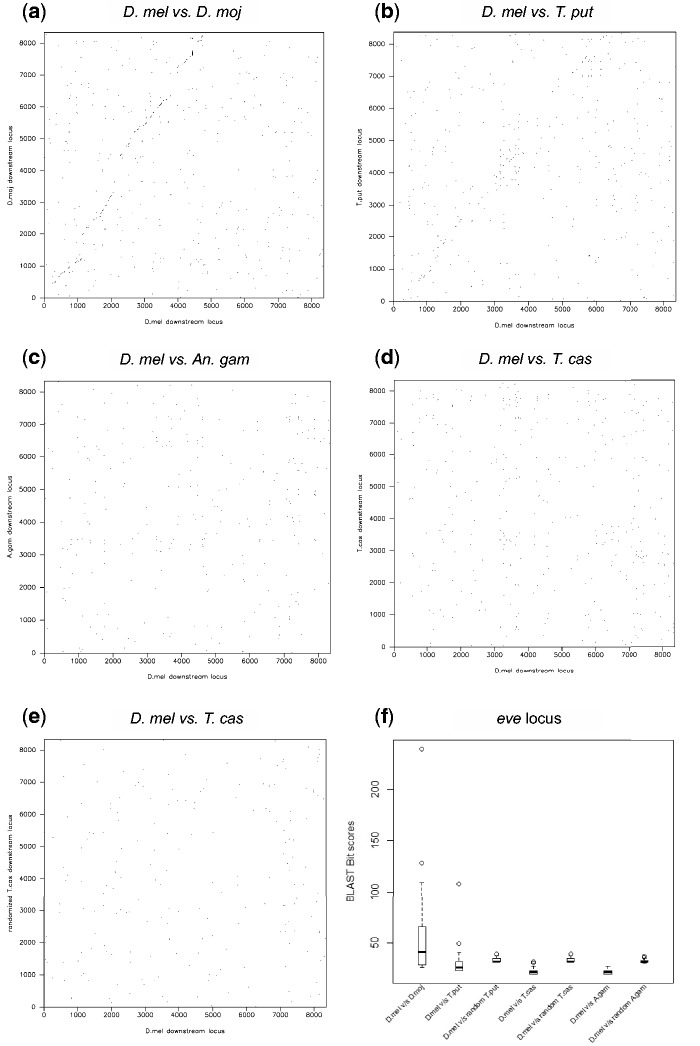
Alignment of intergenic regions of developmental genes between diverged species. Examples in this figure are from the *even skipped (eve)* locus; see supplementary figure S1, Supplementary Material online, for examples from other loci. (*a*) Dotplot alignment of *Drosophila mojavensis* and *D. melanogaster* (∼60 Ma) downstream intergenic regions shows clear alignment (diagonal) between the two species. (*b*) The more diverged Sepsid fly *Themira putris* (∼75 Ma) lies near the edge of noncoding alignment (weak diagonal) as previously reported by Crocker and Erives (2008) and Hare et al. (2008). Neither mosquitoes (*c*) nor beetles (*d*) show recognizable alignment, as can be seen from the absence of a visible diagonal and similarity to alignment with randomized sequence (*e*). The dotplot results are confirmed by BLAST analysis (*f*), which shows that only *D. mojavensis* and *T. putris* have a BLAST score distribution with scores exceeding those obtained from randomized sequence (for *D. mel–T. putris* vs. *D. mel–randomized T. putris*, *P* < 0.024, uncorrected one-sided Student’s *t*-test).

We next sought to determine whether orthologous CRMs could be identified through alignment of noncoding regions surrounding developmental genes of interest. Previous results indicated that even within the Diptera, flies and mosquitoes have diverged too much for most noncoding sequences to align (Engstrom et al. 2007; Sieglaff et al. 2009). We used a sensitive BLAST analysis along with analysis of dot-matrix alignments to look for regions of local alignment in the intergenic regions flanking developmentally important genes with similar expression in *Drosophila* and the other four species. We were unable to align any of the sequences we tested (fig. 3 and supplementary fig. S1, Supplementary Material online). Thus, if CRMs have been conserved, it has been at a level undetectable by sequence alignment. In order to identify the relevant CRMs, therefore, we adapted a method we previously developed for finding functionally related CRMs within the *Drosophila* genome for cross-species CRM discovery.

### Cross-Species Supervised CRM Prediction Pipeline

**Fig. 4.—:**
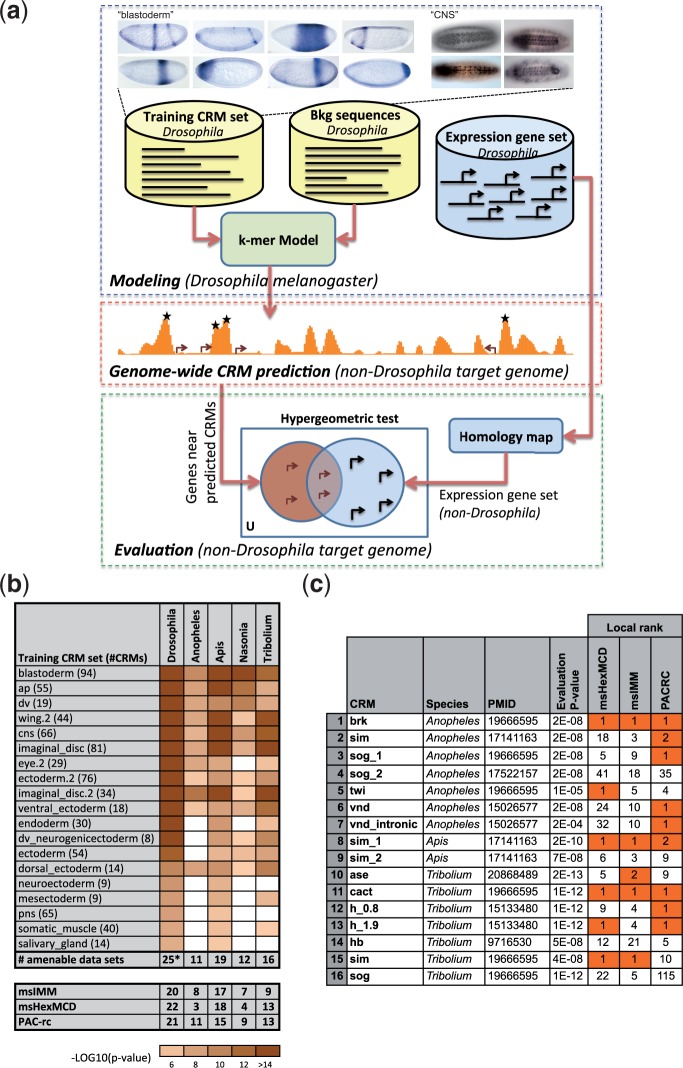
(*a*) Pipeline for cross-species supervised CRM prediction. Top: A set of CRMs that regulate similar gene expression patterns is selected as a training set. Expression driven by CRMs from the *blastoderm* (left) and *CNS* (right) training sets are pictured. Note that there is a range of related but nonidentical patterns. (Blastoderm embryo pictures are adapted from Schroeder et al. [2004] under the terms of the CC-BY license.) A statistical model is then trained on *k-mers* in this training set of CRMs as well as non-CRM sequences from *Drosophila*. Separately, an “expression gene set” is defined as *Drosophila* genes with expression patterns matching the training set. Middle: The trained model is used to scan a non-*Drosophila* target genome, and score every 500-bp window in the genome for similarity to the training set in *Drosophila*. Highest scoring windows (marked with asterisks) are predicted to be CRMs. Bottom: The expression gene set in *Drosophila* is mapped through homology to a gene set (in the target genome) whose expression is expected to be similar to that of predicted CRMs. Genes near predicted CRMs are tested for enrichment in this gene set, providing a preliminary statistical assessment (evaluation *P* value) of the predictions. *Additional data sets amenable to supervised CRM prediction in *Drosophila* are shown in supplementary table S2, Supplementary Material online. (*b*) Data sets amenable to cross-species supervised CRM prediction. Shown are the data sets where an evaluation *P* value ≤ 1E-5 was observed for at least one statistical model, in at least one non-*Drosophila* species. Color intensity of a cell is proportional to the negative logarithm of the evaluation *P* value, with any *P* value ≥ 1E-5 being represented as a white cell. The last row in top panel reports the total number of amenable data sets in each species. The bottom three rows show the number of data sets amenable to cross-species supervised CRM prediction (at the 1E-5 threshold) for each statistical model and each species. More detailed results are shown in supplementary table S2 and figure S2, Supplementary Material online. (*c*) Evaluation of three statistical models on known CRMs in *An. gam*, *A. mel*, and *T. cas*. For each CRM, the local rank over a 100-kb region (430 windows on average) surrounding the relevant gene, under each statistical model, is provided. Cases where local rank is ≤2 are highlighted. The given evaluation *P* value is the best from among the three statistical models. Global ranks for all three models are provided in supplementary table S5, Supplementary Material online.

Our basic approach to CRM prediction (fig. 4*a*) is to use a training set of *Drosophila* CRMs, defined by a common functional characterization (e.g., mesoderm expression), to build a statistical model that captures the short subsequence (*k-*mer) count distribution in these CRMs. An appropriate set of background sequences (e.g., random segments of the noncoding genome) is also used in this training phase. Note that CRMs in the training set regulate related, but often not identical gene expression (fig. 4*a*, top). The trained model is then used to score every 500-bp long window in the “target genome,” and the highest peaks in the resulting score profile are predicted to be CRMs. This “supervised CRM prediction” approach was demonstrated in our previous work (Kantorovitz et al. 2009; Kazemian et al. 2011) to accurately predict CRMs for many different regulatory systems in *D. mel*, using respective training sets from the same species (available in supplementary file S1, Supplementary Material online). We extended this approach to predict CRMs in the four other insect genomes—*An. gam*, *T. cas*, *A. mel*, and *N. vit*—by using the trained models from *Drosophila* CRMs to scan each of these target genomes separately. These genomes are greatly diverged from *D. mel* with estimates of last common ancestors ranging from 250 Ma (*An. gam*) to 350 Ma (*A. mel*) (Wiegmann et al. 2009, 2011). We hypothesized that despite our inability to align noncoding sequences between these genomes, the underlying *cis*-regulatory similarity would be strong enough that the *k-*mer composition of a CRM in the target genome would be significantly similar to that of *Drosophila* CRMs active in similar spatiotemporal domains. On the other hand, if regulatory mechanisms have extensively diverged or have evolved so that completely different sets of TFs are responsible for generating similar expression patterns, we would be unable to recover functionally related CRMs. Three different types of statistical models—fixed order Markov Chain, IMM, and Poisson word count models, respectively, named “msHexMCD,” “msIMM,” and “PAC-rc” following terminology from Kazemian et al. (2011) (see Materials and Methods)—were used independently to predict candidate CRMs that are most similar to each training set in *Drosophila*.

As a preliminary assessment of the viability of our cross-species approach, we tested statistically whether the candidate CRMs in each target genome are enriched near genes with expression patterns (inferred from homology to *Drosophila*) matching those conferred by the training CRMs. Such enrichment would be expected if the candidate CRMs are functionally similar to the training set (fig. 4*a*, “Evaluation”). This was indeed the case, with evaluation *P* values ≤ 1E-5 (Hypergeometric test, see Materials and Methods) observed for 11–19 of 36 different regulatory systems in each species (fig. 4*b* and supplementary table S2, Supplementary Material online), and with 13 data sets exhibiting significant *P* values in at least three non-*Drosophila* genomes. The three methods exhibit complementary strengths, as noted also in Kantorovitz et al. (2009) and Kazemian et al. (2011), with several cases where *P* values on the same data set differ substantially between methods (supplementary fig. S2, Supplementary Material online). We refer to a data set with evaluation *P* value ≤ 1E-5 as “amenable” to prediction under its respective statistical model. Note that although the evaluation *P* values reveal general trends such as which species or which regulatory systems are more amenable to the approach, they do not suggest expected error rates for prediction. They are also likely to underestimate statistical significance both because gene expression patterns in the target genomes were inferred based on homology to *D. mel* genes and because the *P* values are calculated based on the assumption that candidate CRMs regulate their nearest neighboring genes. However, complete gene expression data are often unavailable both for *D. mel* and for the other species, and the correct regulated genes will not always be the closest ones.

It is worth noting that our motif-blind, *k*-mer-based approach has been previously demonstrated as being superior to or comparable to more popular, motif-based methods for CRM discovery in *D. mel* (Kantorovitz et al. 2009; Kazemian et al. 2011). Here, we performed a similar comparison on non-*Drosophila* species for the two data sets (anterior–posterior and dorsoventral patterning) where relevant TFs and motifs are best known. Performance of motif-based methods on both data sets was poor for all non-*Drosophila* target species (supplementary table S3, Supplementary Material online), further supporting our choice of *k-*mer-based methods for cross-species CRM discovery.

### Effective Recovery of Known CRMs in Non-*Drosophila* Species

To facilitate validation of our predictions, we focused our attention on those candidate CRMs that were located near genes with an expression pattern (either known or inferred by orthology to *D. mel*) that matches the training set. As discussed above, although not all orthologs are expressed in related patterns, most of the patterning genes that have been studied have similar expression to their *D. mel* orthologs during at least some developmental stages. We reasoned that restricting our search to the neighborhood of such genes would simplify interpretation of any observed expression because we would accordingly have a reasonable expectation of the expression pattern associated with a candidate CRM. We therefore redefined a candidate CRM, corresponding to a particular statistical model, as one with the following properties: 1) The data set being used is amenable to prediction, 2) the candidate CRM is located within 20 kbp of a gene whose *Drosophila* ortholog is annotated with an expression pattern matching the training set, 3) its score (global rank) is in the top 0.5% of all segments in the target genome, and 4) it is one of the highest scoring segments within 50 kbp to either side of the gene (“local rank” ≤ 2). Approximately 7,100 sequences met these criteria (supplementary table S4, Supplementary Material online). To evaluate this scheme, especially the newly introduced “local rank” criterion, we examined how a set of 16 experimentally verified CRMs in non-*Drosophila* insect genomes, known from the literature, scored with our models (fig. 4*c*). Remarkably, 12 of the 16 bona fide CRMs were ranked at 1 or 2 of approximately 400–500 scored segments in their respective locus (of length 108 kbp on average) by at least one of the models. Once again we noted complementarity among the three models, with each model placing the known CRM in the top two local ranks in 5–9 of the 16 instances. In 7 of the 12 cases with high local rank, the known CRM did not meet the stringent global rank criterion used in our previous work on the *Drosophila* genome (Kantorovitz et al. 2009; Kazemian et al. 2011); however, the global rank was always better than the weaker 0.5% threshold introduced above, justifying our choice of using both local and global ranks to define candidate CRMs (supplementary table S5, Supplementary Material online). The extremely limited number of verified CRMs in the species of interest precludes a more comprehensive in silico evaluation that includes specificity estimates.

### In Vivo Validation of Candidate CRMs

We selected a set of 24 segments, of length 500–1,000 bp, from among the four insect species to test for functional activity through in vivo reporter gene assays (supplementary table S6, Supplementary Material online). Due to the relative difficulty (*T. cas*, *An. gam*) or current inability (*A. mel*, *N. vit*) to make transgenic animals using native hosts, sequences were cloned from their native species and tested in transgenic *Drosophila*. (We note that the few examples of CRMs reported in the literature for these other species similarly have been tested through reporter assays in *Drosophila*.) Roughly two-thirds of these test segments (15/24) were chosen from the candidate CRMs (as defined above); the remainder were chosen to allow assessment of false positives and did not score sufficiently high in terms of global and/or local rank. Overall, the 24 test segments were selected to span a broad range of global ranks (4–60,512 of ∼1 million windows; median = 528) and local ranks (1–50 of ∼430 windows per gene; median = 1) under the three prediction models from ten different training sets. Moreover, the sequences not qualifying as candidate CRMs were chosen from loci for which we were also testing high-scoring candidates. This served as a control for the possibility that any sequence selected from the locus would function as a gene-specific CRM. A detailed characterization of the test set is provided in supplementary table S6, Supplementary Material online.

**Fig. 5.—:**
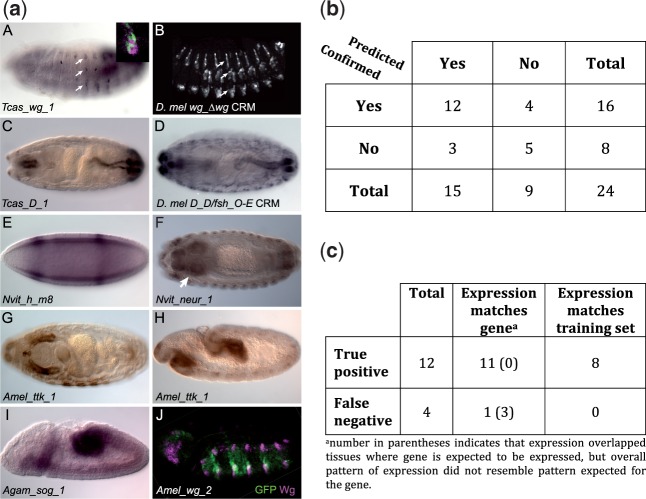
(*a*) Experimentally validated enhancers from diverged arthropods. Predicted CRM sequences were used to drive reporter gene expression in transgenic *Drosophila*. Expression was visualized using immunohistochemistry (*A*, *B*, *C*, *D*, *F*, *G*, *H*, *J*) or in situ hybridization (*E*, *I*). All embryos are shown with anterior to the left. Panels (*A*), (*B*), (*E*), (*H*), (*I*), and (*J*) are lateral views with dorsal to the top; (*C*), (*D*), (*F*), and (*G*) are dorsal views. (*A*) A predicted *T. cas wg* CRM regulates gene expression in a pattern similar to that of the (*B*) *Drosophila wg_Δwg* enhancer (Von Ohlen and Hooper 1997) (compare arrows in [*A*] and [*B*]; panel *B* courtesy of Scott Barolo). Inset in (*A*) shows colocalization of GFP (green) and Wg (magenta) protein expression. (*C*) A predicted CRM for the *T. cas Dichaete* ortholog regulates gene expression in late-stage embryos identical to what is seen with the *Drosophila Dichaete D_D/fsh_O-E* enhancer (panel *D*; Ochoa-Espinosa et al. 2005). GFP expression in the anal ring (partially out of focus in *C*) likely represents perdurance of GFP protein from earlier stages as it is not observed using in situ hybridization. (*E*) A predicted early embryonic stripe CRM for the *N. vit hairy* ortholog gives a two-stripe pattern similar to that of a *Drosophila hairy* CRM (Howard and Struhl 1990) (see also fig. 6*a*). (*F*) A CRM for the *N. vit neuralized* gene drives expression in the central and peripheral nervous systems; arrow denotes the brain (see also supplementary fig. S3, Supplementary Material online). (*G*) A CRM for the *ttk* gene from *A. mel* regulates expression in the salivary gland and (*H*) the developing midgut; additional expression is shown in supplementary figure S3, Supplementary Material online. (*I*) A CRM predicted for *An. gam sog* regulates expression in the mesoderm during germ band extension. (*J*) A “false negative” result is obtained when a sequence not predicted to be a CRM, here from the *A. mel wg* locus, drives patterned gene expression (GFP expression, green). Note that unlike what we see with our predicted *T. cas wg* CRM (panel *C*), reporter gene expression in this case is not confined to Wg-expressing cells (magenta). (*b*) Summary of results from in vivo testing of 24 segments. Each segment is characterized as being a predicted CRM or not, and whether reporter activity was confirmed or not. (*c*) Summary of lines with positive expression. Shown are the numbers of lines with expression pattern matching to the gene or covered by one or more high scoring training sets.

The experimental assays described above confirmed reporter gene activity for 16 CRMs in four non-*Drosophila* insect genomes, nearly doubling the number currently described (fig. 4*a* and supplementary fig. S3 and table S6, Supplementary Material online). Of these, 12 (75%) were among the sequences “predicted” by one of the models and are thus considered to be true positive results; the remaining four are considered false negative results (see 2 × 2 confusion matrix in fig. 5*b*). Three of the 15 candidate CRMs failed to drive gene expression in our reporter assay and are considered to be false positive results. Thus, in the selected set of 24 segments, we observed a precision of 80% and a recall of 75%, with an F1 score, which is the harmonic mean of precision and recall, of 77%. The accuracy of predicting negatives was lower, with specificity equaling 63%. Requiring a predicted CRM to meet the above-mentioned criteria from two statistical models rather than one led to an improved specificity of 75% with a slightly lower F1 score of 71% (see supplementary table S7, Supplementary Material online). Surprisingly, given the large evolutionary distances between *D. mel* and the other insects for which we predicted CRMs, the observed success rates are only moderately lower than what we have previously demonstrated for using *Drosophila* training data to predict CRMs in the *Drosophila* genome.

Each of the 12 true positives drove an expression pattern commensurate with the known or inferred (from homology to *Drosophila*) expression of its likely associated gene (see supplementary table S6, Supplementary Material online). This is significant, as each training set contains a range of similar, but not identical, expression patterns. Coincidental activity due to similarity in *k*-mer profile between a candidate CRM and the training set would therefore not be expected to precisely match a specifically chosen gene’s expression. In contrast to the true positives, only one of the four false negatives, that is, test segments that drove expression but did not meet the criteria for candidate CRMs, clearly matched the expression of the associated gene. The remaining three may partially overlap, but not fully encompass, regulatory sequences (fig. 5*a*.*J* and *c* and supplementary fig. S3, Supplementary Material online). Furthermore, for 8 of the 12 true positive predictions (75%), the reporter gene expression pattern we observed fits at least one high-scoring training set (fig. 5*c*; if a segment meets the criteria of a candidate CRM as per a given model, the training set on which the model was built is called a “high-scoring training set” for that segment). This suggests that the statistical models were able to learn not only general features of CRMs but also features pertaining to the expression pattern associated with the training set.

### Highly Related Expression Patterns Suggest Identification of Homologous CRM Pairs

**Fig. 6.—:**
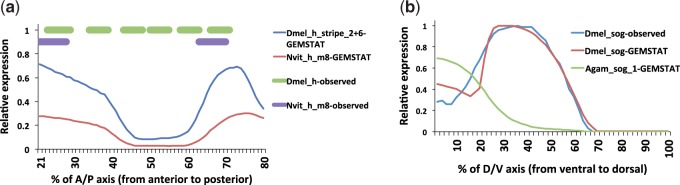
(*a*) Modeled gene expression regulated by *hairy* CRMs in *D. mel* (“*Dmel_h_stripe_2+6*,” *construct ET15* of Howard and Struhl [1990]) and *N. vit* (“*Nvit_h_m8*,” this study)*.* Expression mediated by the *N. vit hairy* CRM (*Nvit_h_m8*-observed) has an anterior stripe that starts slightly anterior to the endogenous *D. mel h* stripe 1 (“*Dmel_h*-observed,” leftmost green bar) and ends just before the posterior margin of the stripe, whereas the posterior stripe begins immediately posterior to the endogenous stripe 5 and extends almost to the posterior margin of stripe 6 (“*Dmel_h*-observed,” rightmost green bar). The GEMSTAT model predicts similar expression profiles for both CRMs (*Dmel_h_stripe_2+6*-GEMSTAT, *Nvit_h_m8*-GEMSTAT), with a posterior stripe overlapping the endogenous *hairy* stripe 6 and a broad anterior domain that straddles endogenous *D. mel* stripes 1 and 2. (*b*) Modeled gene expression regulated by *sog* CRMs in *D. mel* (“*Dmel_sog_shadow*”; Hong et al. 2008) and *An. gam* (“*An. gam_sog_1*,” this study)*.* The endogenous expression pattern of *sog* in *D. mel* is ectodermal (blue) and agrees with the GEMSTAT-predicted profile (red) for the *Dmel_sog_shadow* CRM. The same model predicts a mesodermal expression pattern as output of the *Agam_sog_1* CRM as reported in this work.

In several cases, the reporter gene expression not only matches the expression pattern of the putative associated gene but also closely resembles the activity of a known *Drosophila* CRM from the orthologous locus. For instance, the activity of a *T. cas wg* CRM is like that of the *wg_Δwg* enhancer (Von Ohlen and Hooper 1997) (*Tcas_wg_1*; fig. 5*a*.*A*;), whereas a *T. cas Dichaete* CRM recapitulates postblastoderm expression driven by the *D_D/fsh_O-E* enhancer (Ochoa-Espinosa et al. 2005) (*Tcas_D_1*; figs. 5*a*.*C* and 4*a*.*D*). Similarly, an *N. vit* CRM located in the *N. vit hairy* locus drives gene expression in two stripes in *Drosophila* embryos reminiscent of two known *D. mel* CRMs: *h_stripe_6+2 (ET15* in Howard and Struhl [1990]) and *h_ET5* (expressing in *h* stripes 1 and 5) (*Nvit_h_m8;* fig. 5*a*.*E*; Howard and Struhl 1990). A comparison between the *N. vit* CRM and the *Drosophila h_stripe_6+2* CRM indicates an overall similarity of binding site composition (supplementary fig. S4*a*, Supplementary Material online). To further explore this similarity, we used a quantitative model (GEMSTAT for A/P CRMs, see Materials and Methods) to predict the expression driven by the two sequences. Encouragingly, the model predicts highly similar expression readout from the two sequences, with a pattern that closely matches that observed for the *N. vit* CRM (fig. 6*a*). Thus, despite a complete lack of nucleotide-level alignment (see below), these beetle and wasp CRMs appear to be homologous to their *Drosophila* CRM counterparts: they are drawn from orthologous loci, have related binding site composition (see below), and share common regulatory activity when tested in the same transregulatory context.

### A CRM from An. gam sog Maintains the Mosquito, Rather Than the Fly, Expression Pattern

Of particular note is a CRM discovered in the *An. gam sog* locus. *Drosophila sog* is expressed in the neurogenic ectoderm and mesectoderm, resolving primarily to the mesectoderm by mid germband extension. In contrast, the *An. gam sog* CRM drives reporter gene expression in the mesoderm (*An. gam_sog_1;* fig. 5*a*.*I*). This pattern is consistent with the described expression of *An. gam sog*, which is mesodermally, not ectodermally, expressed, perhaps due to utilization of a different combination of *cis*-regulatory inputs, or of the same inputs with different strengths, by the responsible CRM (Goltsev et al. 2007). This is supported by our GEMSTAT modeling, which predicts ectodermal and mesodermal expression domains for the *D. mel* and *An. gam* CRMs, respectively (fig. 6*b*). Importantly, the candidate CRM sequence from *An. gam* retains *An. gam sog* regulatory activity even when placed into the *D. mel* genome. This provides one of the relatively few known examples of regulatory evolution at the *cis-*level that has led to qualitative (Jeong et al. 2008) rather than quantitative (Wunderlich et al. 2012) expression divergence.

### CRM Pairs Exhibit Significant Similarity at the TF Binding Site Level, but Not at the Sequence Level

**Fig. 7.—:**
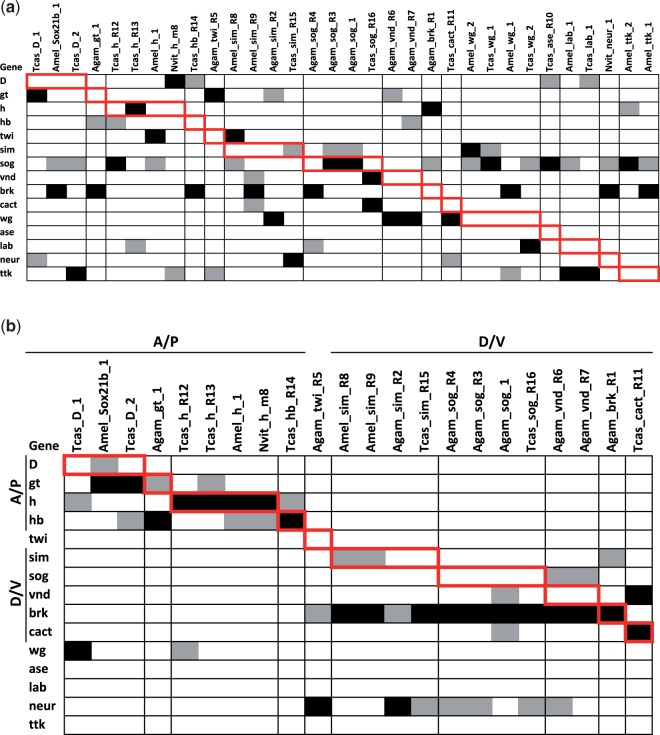
Measures of sequence and motif similarity between CRMs. (*a*) Absence of alignment-based similarity. For each non-*Drosophila* CRM (columns) and each *Drosophila* gene locus (rows), defined as 20 kb on either side of the gene plus introns, we recorded the best LASTZ HSP score between the CRM and the gene. The two highest scoring genes for each CRM are shown in black and gray. For each CRM (column), the *Drosophila* ortholog of the regulatory target of the CRM is indicated by red borders. Note that only 4 of 32 CRMs are mapped by LASTZ to the expected gene locus (shaded cells with red border, Binomial test *P* value = 0.63). CRMs identified in this study are named as in supplementary table S6, Supplementary Material online. Previously known CRMs are named ending in _Rn, where “n” refers to the row number of the corresponding CRM in fig. 4*c*. (*b*) Evidence for similarity of motif composition. Each non-*Drosophila* CRM is scored by its best matching sequence window in each *Drosophila* gene locus, defined as in panel (*a*), using the Regulus similarity score. Only CRMs related to A/P or D/V patterning were examined as many of the relevant motifs are known for these. The two highest scoring genes for each enhancer are shown in black and gray, and a red border indicates the gene expected to harbor a homologous CRM. Note that 11 of 22 enhancers are mapped by Regulus to the expected gene locus (Binomial test *P* value = 4 × 10^−5^). CRM names are as in panel (*a*).

We showed above that noncoding regions are not alignable between *Drosophila* and the other studied species for a subset of orthologous loci (fig. 2 and supplementary fig. S1, Supplementary Material online). To ensure that our newly identified CRMs did not represent exceptions to this, we used LASTZ (Harris 2007) to test for conserved segments between each newly verified non-*Drosophila* CRM and a 20-kbp region on either side (plus introns) of the orthologous *Drosophila* target gene. The results, consistent with our findings in figure 2 and supplementary figure S1, Supplementary Material online, show that alignment between related pairs of CRMs is not distinguishable from that between unrelated pairs of sequences (fig. 7*a*; also see supplementary note S2, Supplementary Material online). We then asked whether related sequence pairs have significant similarity in terms of their putative binding site content, which is not detectable with nucleotide-level alignments. To this end, we designed two different measures of pairwise CRM similarity. These measures are distinct from the similarity scores used above for predicting CRMs, which compare a candidate CRM with a set of similar CRMs. As *k-*mer-based statistics have not been shown to accurately capture pairwise CRM similarity, the two new measures were designed to use binding site motifs, and we used them in the context of CRMs related to the anterior–posterior or dorsal–ventral patterning systems, for which several relevant motifs are known. The first measure casts each sequence as a vector of motif scores and compares two vectors using the cosine similarity measure (see supplementary note S3, Supplementary Material online, titled “MoCS”). This score was unable to discriminate related sequence pairs.

The second measure, called Regulus, is a probabilistic score that reflects the number of binding sites common to both sequences, while properly accounting for variable strengths of a TF’s binding sites and variable frequencies of different TFs’ sites (see Materials and Methods). To our knowledge, there is no obvious comparator in the literature for Regulus as a similarity score of two CRMs based on their motif compositions. (The obvious approach of counting every motif’s occurrence in either CRM and comparing these count vectors was explored in the MoCS score mentioned above, and its variants that are not reported here.) The Regulus score was able to map the non-*Drosophila* CRMs to the correct *Drosophila* gene locus with an accuracy of 50% (11 of 22 CRMs mapped correctly, Binomial test *P* value = 4 × 10^−^^5^, fig. 7*b*), compared with the 18% accuracy (4 of 22, *P* value = 0.34) on the same set of CRMs when using LASTZ as the similarity measure.

**Fig. 8.—:**
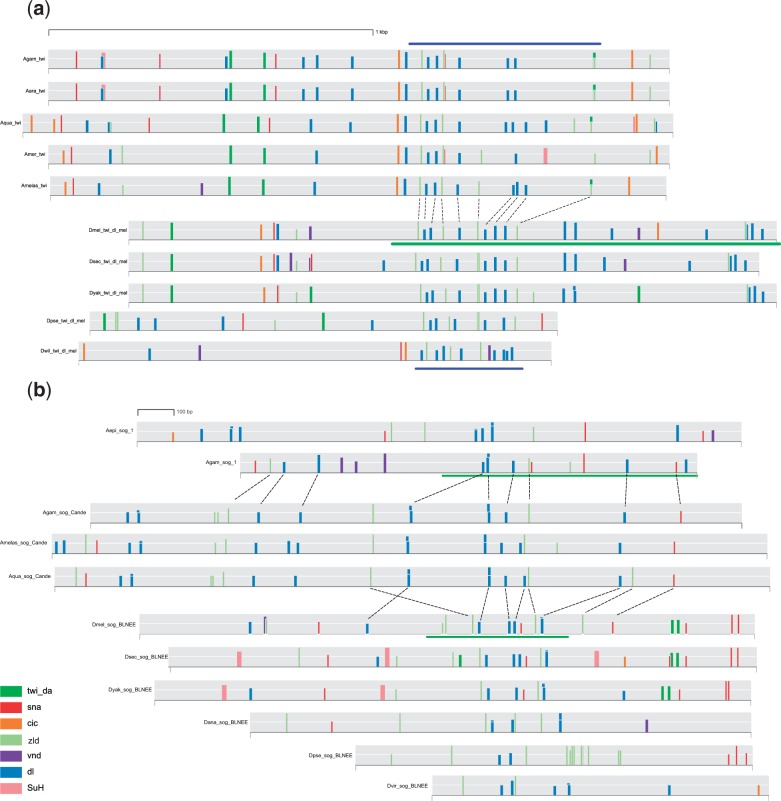
TF binding site motifs in functionally related CRM pairs. Binding site motifs are indicated by vertical colored bars, with bar heights correlating to degree of match to the motif. Horizontal green bars indicate the extent of the sequences tested in vivo. Dashed lines indicate motif similarities among nonorthologous CRMs or CRMs from different genera. For clarity, only a subset of conserved motifs are marked. (*a*) Motif alignment of the *D. mel* “*twi_dl_mel*” *twi* CRM and the orthologous sequence from four other Drosophilids with the *An. gam twi* CRM from Cande, Goltsev, et al. (2009). Blue bars highlight the similar “core” motif arrangement of sites for DORSAL lying between sites for ZELDA. Note the conserved arrangement of motifs as evidenced by lack of crossing of the dashed lines. (*b*) *sog* CRMs from *Drosophila* and *Anopheles*. The *An. gam sog* CRM from Cande, Goltsev, et al. (2009) is shown in the middle, aligned with two orthologous *Anopheles* sequences. The entire pictured sequence was confirmed in vivo. Aligned sequences from the *Drosophila sog_broad_lateral_ectodermal_enhancer* (BLNEE) are shown at the bottom. Alignments at the top represent the *An. gam_sog_1* CRM (this study, fig. 5*I*) from *An. gam* and *An. epiroticus*. Close motif alignment can be observed between all three sets of CRMs, and for the CRM from Cande, Goltsev, et al. (2009) and its *Drosophila* counterparts in particular. Species abbreviations: *Agam*, *Anopheles gambiae; Aara*, *An. arabiensis; Aepi*, *An. eprioticus; Aqua*, *An. quadriannulatus; Amer*, *An. merus; Amelas*, *An. melas; Dmel*, *Drosophila melanogaster; Dsec*, *D. sechellia; Dyak*, *D. yakuba; Dpse*, *D. pseudoobscura; Dana*, *D. ananassae; Dwil*, *D. willistoni; Dvir*, *D. virilis*.

To help us better interpret the Regulus results, and to further explore the binding site content of the CRMs, we made a graphic map of the identified motifs along the sequences for several pairs of CRMs (from this study as well as from the literature; fig. 8 and supplementary fig. S4*a–e*, Supplementary Material online). Because multiple species of flies, bees, and mosquitoes have now been sequenced, we included orthologous regions (based on alignment within a given subfamily or genus) of the CRMs for each of the respective *D. mel*, *An. gam*, and *A. mel* CRMs where possible. These multispecies alignments help to focus attention on the most well-conserved motifs and bring potentially important patterns of motif distribution into better relief. Consistent with the results from Regulus, we observed similarity in overall motif makeup within each pair of CRMs. Moreover, despite the complete lack of alignable noncoding sequence between genera (supplementary fig. S1, Supplementary Material online, and data not shown), we could in many cases identify a rough “core” region with a loose linear correlation of motifs. This was particularly apparent between the more closely related flies and mosquitoes for the early embryonic *twi* and *sog* CRMs, which share regions of heavily interspersed DORSAL and ZELDA motifs (fig. 8), and the *brinker* (*brk*) CRMs, with DORSAL/SNAIL/ZELDA regions (supplementary fig. S4*b*, Supplementary Material online). We observed a striking correspondence in motif pattern between our newly discovered *An. gam sog* CRM (*Agam_sog_1*), the *An. gam sog* CRM previously described by Cande, Goltsev, et al. (2009), and the *D. mel* CRMs *sog_broad_lateral_neurogenic_ectoderm* and *sog_NEE/sog_shadow* (fig. 8*b* and data not shown). Similarly, a clear motif consensus could be seen among the CRMs driving early expression of *sim* in flies, mosquitoes, and bees, with the CRMs from all three families having a core of DORSAL, ZELDA, and SNAIL sites (supplementary fig. S4*c*, Supplementary Material online). Although a clearly defined core pattern could not be defined for comparisons with the *N. vit h* and *T. cas D* CRMs, an overall resemblance of motif composition and arrangement is still observed (supplementary fig. S4*a* and *d*, Supplementary Material online).

Taken together, our motif-level analysis, along with our observations on the functionally homologous pairs of CRMs above (for *sog*, *wg*, *h*, and *D*), strongly indicates that CRMs may diverge well beyond recognition by nucleotide-level alignment methods while still maintaining their overall *cis*-regulatory logic, at least in part through similar motif composition.

## Discussion

### Are the Candidate CRMs True CRMs?

Our candidate CRM sequences were selected due to *k-*mer profile similarity to known *Drosophila* CRMs. A trivial interpretation of the results, therefore, would be that because the candidates have a fly-like *k-*mer profile, they drive fly-like reporter gene expression without being true regulatory elements in their native species. Although virtually all currently identified insect CRMs were defined through validation by reporter gene assays in transgenic *Drosophila* (e.g., Wolff et al. 1998; Erives and Levine 2004; Markstein et al. 2004; Zinzen et al. 2006; Goltsev et al. 2007; Cande, Chopra, et al. 2009; Cande, Goltsev, et al. 2009; Ayyar et al. 2010), and *Drosophila* reporter gene assays have even been used to demonstrate deep regulatory homologies between fly and mouse CRMs (Awgulewitsch and Jacobs 1992; Malicki et al. 1992; Xu et al. 1999), the only definitive way to rule out this possibility would be to conduct reporter gene assays in each individual species (which is not currently possible for *A. mel* and *N. vit*). Nevertheless, there is considerable evidence to support the view that the identified candidates represent true CRMs in their respective species. In particular, a substantial number of the CRMs drove reporter gene expression not only in the generally expected pattern (e.g., blastoderm, peripheral nervous system) but also in the specific pattern expected for the associated gene. Indeed, in several cases the CRM-driven expression closely resembled that regulated by known *Drosophila* CRMs from the orthologous locus. Although it is conceivable that sequences coincidentally matching the training set’s *k-*mer profile may exist and drive generally related gene expression, note that each training set encompasses a range of nonidentical expression patterns. For instance, the *blastoderm* training set includes genes expressed at different positions within the blastoderm embryo, genes expressed in either of the A/P or D/V axes, and both gap-like and pair-rule-like patterns. Similarly, the *cns* data set includes genes expressed in the brain, the lateral portion of the ventral nerve cord, and the midline of the ventral nerve cord. Given this diversity in expression pattern even within a training set, we expect that although a coincidental “CRM-like” sequence might drive expression that resembles some aspects of the training set patterns, it is highly unlikely—especially given the observed lack of sequence conservation—that such sequences would be maintained in an orthologous locus, drive the expression pattern expected for the orthologous gene, and recapitulate the regulatory activity of a specific *D. mel* CRM associated with that gene. Thus, the *Nvit_h_1* CRM drives a two-stripe *h*-like pattern rather than, say, a broad posterior gap gene pattern. Furthermore, the *An. gam sog* CRM we identified drove gene expression not in the *D. mel sog* pattern, but in the *An. gam sog* pattern similar to expression from other known *An. gam sog* CRMs. This strongly suggests that the sequence represents a true *An. gam sog* CRM. Collectively, these data provide a compelling argument in favor of the candidate CRMs being bona fide regulatory elements in their own species, and not merely emulating *Drosophila* CRMs by virtue of coincidental sequence similarities.

### Cross-Species CRM Prediction

One goal of this work was to determine whether *Drosophila* data could be used to predict CRMs in highly diverged species, on a genomic scale; previous cross-species predictions have only been performed on a locus-by-locus basis (e.g., Cande, Goltsev, et al. 2009). In our previous work (Kantorovitz et al. 2009; Kazemian et al. 2011), we reported on extensive comparisons among different approaches to within-species supervised CRM prediction in *Drosophila*. We also evaluated the complementary strategy of identifying TF motifs overrepresented in the training CRMs, using a collection of known motifs for *Drosophila*, followed by motif-based CRM prediction using HMM models (Frith et al. 2003; Sinha et al. 2003). The lesson from these evaluations was that our three scores for supervised CRM prediction—msHexMCD, msIMM, and PAC-rc—were equivalent or superior to any other methods available at the time, including more popular methods based on motif matches. (We reconfirmed the advantage over motif-based methods in this work.) Hence, using these three scores for cross-species prediction was a natural choice, and we leave the assessment of other *k-*mer-based statistical scores (Leung and Eisen 2009; Lee et al. 2011; Ren et al. 2013) on this new problem as future work. We also note that the potential of *k-*mer-based scores in cross-species CRM characterization was illustrated by Peterson et al. (2009), who used aggregates of short word matches to assign orthology relationships to pairs of validated CRMs in the *eve* locus between *D. melanogaster* and tephritid (true fruit fly) genomes.

Cross-species prediction presents special challenges for evaluating results, as the expression pattern of CRM target genes in the native organism is frequently not known, and even known expression patterns cannot always be mapped cleanly between different organisms. In choosing candidates for validation here, we attempted to focus on genes whose expression patterns were expected either to be reasonably well-conserved, or previously described. Although alterations in gene expression pattern between species complicate evaluation, we expect that our methodology will nevertheless prove robust to such changes, as evidenced by our successful prediction of the *An. gam sog* mesodermal CRM. Altered expression due to changes in the distribution of transacting factors should of course have no effect on our sequence-based supervised prediction. However, as our method scores the statistical distribution of *k*-mers and not direct counts or identification of specific binding sites, it should also be able to predict CRMs which drive an altered pattern of gene expression due to gain or loss of a specific TF but which integrate an overall similar set of regulatory inputs.

### Direct Homology versus Convergent Evolution

Previous studies of dorsal–ventral patterning in non-Drosophilid insects point to weakly conserved regulatory mechanisms with a high degree of turnover and exchange of binding sites (Erives and Levine 2004; Zinzen et al. 2006; Goltsev et al. 2007; Cande, Goltsev, et al. 2009). A small number of identified CRMs regulating later embryonic development of the heart also support the idea of conserved regulatory mechanisms and homologous CRMs (Cande, Chopra, et al. 2009). Very few data exist with respect to patterning in the anterior–posterior axis. CRMs have been identified for *T. cas hunchback* (*hb*) and *hairy* (*h*) (Wolff et al. 1998; Eckert et al. 2004), but although both drive appropriate expression in *Drosophila* embryos, suggesting that the basic *cis–trans* regulatory mechanisms have been conserved, neither has been characterized at the binding site level. A 4-kb noncoding fragment from the *A. mel gt* locus containing binding sites for upstream factors such as Cad, Hb, and Otd drives expression in *Drosophila* in a pattern consistent with *A. mel gt* expression (Wilson and Dearden 2011), but the large size of the fragment makes it difficult to draw definitive conclusions about CRM composition.

Our newly identified CRMs, like many of the previously known non-*Drosophila* CRMs, reside in similar positions relative to their associated genes as do their *Drosophila* counterparts, and we have shown for several of these CRM pairs—in particular, for *sog* and *twi*—strikingly similar TF binding site motif composition and arrangement. (The presence of shared binding sites in short blocks of sequence conservation between otherwise diverged CRM sequences was also noted in Hare et al. [2008].) Overall, this suggests a mechanism of direct evolutionary descent with various degrees of binding site turnover, gain or loss of additional binding sites, and/or changes in expression of TFs accounting for species-specific differences in regulatory activity. These mechanisms have been observed before (e.g., Williams et al. 2008), but generally not over evolutionary distances as vast as those considered here. Taher et al. (2011) were able to identify a number of putatively directly related CRMs between distant species by performing “conservation tunneling” in which they were able to detect alignments between each species and an intermediate species even in the absence of alignable noncoding sequence between the two more distant species (i.e., human–frog, frog–zebrafish alignments to detect human–zebrafish-related CRMs). However, whole-genome sequences of appropriately positioned insect species to allow such an approach are not currently available, and the rapid radiation of the Insecta makes it questionable whether such intermediate species could be found. Moreover, as noted earlier, the fly–honeybee sequence divergence appears to be more extensive than the human–fish divergence, despite the shorter calendar years (vs. generations) of separation (Zdobnov and Bork 2007).

Nevertheless, given the lack of alignable sequences between the homologous pairs of CRMs, we cannot rule out convergent evolutionary mechanisms. Convergent evolution has been implicated in a number of examples, such as the de novo emergence of the *Pomc* neuronal CRMs in the mammalian lineage (Domene et al. 2013) and the identification of several cross-functional urochordate and vertebrate regulatory sequences by Doglio et al. (2013). The CRM compositions we observe could also represent a mix of direct descent and convergent changes, as has been suggested for CRMs for the *shavenbaby* gene in several *Drosophila* lineages (Frankel et al. 2012). Similarity of motif compositions between functionally similar CRMs, while presumed above as evidence of shared descent, may also result from parallel evolution, if for instance the “solution space” of a specific regulatory functionality is limited and necessitates a unique combination of binding sites (Crocker and Erives 2008). Additional complications may arise when considering “shadow enhancers,” that is, functionally redundant CRMs for the same gene (Hong et al. 2008), believed to confer robustness of expression readouts (Frankel et al. 2010). It is possible for such redundant pairs (or sets) of CRMs to arise by convergent evolution, for example, the *Pomc* neuronal CRMs mentioned above (Franchini et al. 2011), or to exhibit differences in binding site compositions, for example, the *sog* NEE and LSE enhancers (Markstein et al. 2002). This in turn complicates assessing divergent versus convergent evolution in functionally similar CRMs from two species. Much more extensive CRM discovery covering a fuller range of intermediate species and a more comprehensive coverage of each locus will be needed to begin addressing these questions—something the approach we introduce here will be able to facilitate. For a more elaborate discussion of different modes of CRM evolution, we refer the reader to Robinson et al. (2011) and the associated journal issue.

Taken as a whole, the results from our cross-species, motif-blind supervised CRM prediction and motif-based comparison suggest that we have been able to discover genuine CRMs in four diverged insect species, at least some of which share function with their *Drosophila* counterparts. Importantly, our high rate of success in cross-species supervised CRM prediction suggests that the large and ever-growing amount of available experimental data on *Drosophila* CRMs can be leveraged effectively to provide regulatory element annotation for the many insect genomes that have been, or are in the process of being, sequenced. Our results argue strongly that despite extensive binding site turnover and overall sequence divergence, similar regulatory mechanisms govern developmental gene expression even over distances of >350 Myr, and suggest that gene regulatory networks have been directly conserved. Our work provides a possible way forward for detection of and reasoning about regulatory DNA homology, analogous to but going beyond the highly successful BLAST-based paradigm for coding sequences. This will involve the powerful combination of alignment-free methods to predict putative homologs in the orthologous gene locus and experimental methods to characterize the expression readout of the predicted homolog. Complementing the computational tools with experimental validation will likely be necessary, especially for large evolutionary distances, to discern functional homology from the superficial similarity of two sequences that share some common inputs (TF binding sites) but distinct outputs. Depending upon the particular species and CRMs of interest additional computational methods may be of assistance, including alignments of either or both of the remote homologs with their respective “close relatives” (such as in fig. 8), quantitative modeling of expression readout from CRM sequence (such as in fig. 6), and motif-based comparisons (such as with Regulus, or similar tools of the future) if possible. Whole-genome chromatin state profiles (Celniker et al. 2009) and massively parallel enhancer assays (Arnold et al. 2013) will also play an important role in such an endeavor. Our work also highlights some of the subtler challenges in this paradigm, such as the problem of defining or detecting homology of CRMs in the face of potentially diverged trans environments; the *An. gam sog* CRM is a case in point. As the paradigm matures and additional related CRMs from the growing number of sequenced insect genomes become identified, we will be able to trace the evolutionary history of these diverged regulatory networks and determine the various direct and convergent evolutionary influences that shaped the developmental genetics of the vast insect radiation.

## Supplementary Material

Supplementary files S1–S3, notes S1–S4, figures S1–S4, and tables S1–S9 are available at *Genome Biology and Evolution* online (http://www.gbe.oxfordjournals.org/).

Supplementary Data
