# Supplementary material for: Evidence for Deep Regulatory Similarities in Early Developmental Programs across Highly Diverged Insects
Source: Genome Biol Evol. 2014 Aug 29;6(9):2301–20. doi: 10.1093/gbe/evu184 (PMC4217690; doi:10.1093/gbe/evu184)
Supplement: Supplementary Data [file supp_6_9_2301__index.html]

Evidence for Deep Regulatory Similarities in Early Developmental Programs across Highly Diverged Insects — Supplementary Data 

# Evidence for Deep Regulatory Similarities in Early Developmental Programs across Highly Diverged Insects

## Supplementary Data

file

**Files in this Data Supplement:**

- Supplementary Data - zip file
