# Supplementary material for: Evidence for Deep Regulatory Similarities in Early Developmental Programs across Highly Diverged Insects
Source: Genome Biol Evol. 2014 Aug 29;6(9):2301–20. doi: 10.1093/gbe/evu184 (PMC4217690; doi:10.1093/gbe/evu184)
Supplement: Supplementary Data [file supp_evu184_evu184-suppl_data.zip › Supplemental File 3.pdf]

# Regulus

July 16, 2013

## 1 Basic components of probabilistic model

The probabilistic model of Regulus assigns a likelihood score to a pair of sequence $x$ and $y$, given a set of motifs $\{m_i\}_i$. In this section we define various terms and notations used in formally describing this likelihood score, which we will describe in the next section.

The symbol $s$ denotes a sequence (either $x$ or $y$), and $m$ denotes a motif. Also provided is a vector of "candidate sites" of motif $m$ on sequence $s$:

$$w_m^s = (w_m^s[k])_k$$

Here, each $w_m^s[k]$, $k = 1, 2, \ldots$ denotes a candidate site of motif $m$ in sequence $s$, predicted using a pre-determined threshold on LLR scores of PWM matches. The LLR score threshold was chosen to correspond to a p-value of 0.005.

The computationally predicted candidate sites $w_m^s$ may include spurious binding sites. The model probabilistically selects a subset of all candidate sites for use in calculations. Define a Boolean array representing the selected sites of motif $m$ in sequence $s$:

$$t_m^s = (t_m^s[k])_k$$

Let the set of all nucleotide positions outside any motif site on sequence $s$ be denoted by:

$$nw^s$$

Define a vector representing selected candidate sites over all motifs ($m$) and over the two sequences ($s$):

$$t = ((t_m^s)_m)_s = (t^s)_s = ((t_m^s)_s)_m = (t_m)_m = ((t_m^s))$$

Define probability of selecting (versus not selecting) a candidate site of motif $m$ in sequence $s$:

$$p_m^s$$

This is also called the "transition probability" of entering motif state $m$ from any other state when generating sequence $s$.

Define emission probability of site $w$ in sequence $s$ based on the PWM of motif $m$ :

$$e_m^s(w)$$

Define "shell score" of site $w$ corresponding to motif $m$ in sequence $s$ (for use in derivations):

$$h_m^s(w) = p_m^s$$

Define "whole score" of site $w$ corresponding to motif $m$ in sequence $s$ (for use in derivations):

$$v_m^s(w) = h_m^s(w)\, e_m^s(w) = p_m^s e_m^s(w)$$

If a nucleotide does not fall within any selected site, the model is said to enter the "background state" at that position in the sequence. Denote the transition probability of entering the background state at any position in sequence $s$ by:

$$p_{bg}^s$$

Define emission probability of nucleotide position $j$ based on the background frequencies in sequence $s$:

$$e_{bg}^s(j)$$

Define emission probability of a set $w$ of nucleotide positions based on the background frequencies in sequence $s$:

$$e_{bg}^s(w) = \prod_{j \in w} e_{bg}^s(j)$$

Define "shell score" of set $w$ of nucleotide positions in sequence $s$ where the model enters background state (for use in derivations):

$$h_{bg}^s(w) = \prod_{j \in w} p_{bg}^s$$

Define "whole score" of set $w$ of nucleotide positions where the model enters background in sequence $s$ (for use in derivations):

$$v_{bg}^s(w) = h_{bg}^s(w)\, e_{bg}^s(w) = \prod_{j \in w} p_{bg}^s \prod_{j \in w} e_{bg}^s(j) = \prod_{j \in w} p_{bg}^s e_{bg}^s(j)$$

The product of transition probabilities of all states entered by the model in making a site selection $t$ is called the "Base energy term" corresponding to selection $t$:

$$\psi(t) = \prod_s \left( h_{bg}^s(nw^s) \prod_m \prod_k h_{t_m^s[k]?m:bg}^s\left(w_m^s[k]\right) \right) \tag{1}$$

We define the number of shared sites of motif $m$ in selection $t$ as the minimum of the number of selected sites of $m$ over the two sequences:

$$n_m(t) = \min_s\left(\left|\{k | t_m^s[k] = 1\}\right|\right)$$

In other words $n_m(t)$ is the smaller of the two counts of $m$ in the site selection $t$ made over sequences $x$ and $y$.

Contribution of motif $m$ to "similarity weight" of selection $t$:

$$\phi_m(t) = \phi^{n_m(t)}$$

where $\phi$ is a constant.

Similarity weight of selection $t$ is the product of contributions from all motifs:

$$\phi(t) = \prod_m \phi_m(t)$$

Probability of emitting the two sequences, denoted by $S = \{x, y\}$, under selection $t$:

$$P(S|t) = \prod_{s \in S} \left( e^s_{bg}(nw^s) \prod_m \prod_k e^s_{t^s_m[k]?m:bg}(w^s_m[k]) \right) \tag{2}$$

## 2 Models and LLR values

We define two null models $nA$ and $nB$, and a homology model $M$, as shown below.

In null model $nA$, every nucleotide of either sequence $s$ is emitted as per the background frequency distribution, i.e., the normalized frequencies of nucleotides in sequence $s$. Probability score of the two sequences under null model $nA$:

$$P(S|nA) = \prod_s e^s_{bg}(s) \tag{3}$$

In null model $nB$, each selection $t$ of candidate sites in the two sequences $S$ is made with probability *proportional* to $\psi(t)$ defined in the previous section. Then, conditional on the selection $t$ the sequences themselves are emitted with probability $P(S|t)$. Probability score of the two sequences under null model $nB$:

$$P(S|nB) = \sum_t P(S|t) P(t|nB) = \sum_t P(S|t) \frac{\psi(t)}{\sum_t \psi(t)} = \frac{\sum_t P(S|t)\psi(t)}{\sum_t \psi(t)} \tag{4}$$

We note that null model $nB$ is equivalent to a zeroth order HMM that independently generates the two sequences in $S$. In generating either sequence $s$, the HMM sequentially enters either the background state or one of the motif states (there is one state corresponding to each motif), and emits (respectively) either a single nucleotide from the background distribution or a site by sampling from the appropriate motif. Also note that since $\psi(t)$ represents the probability of a state path in an HMM, the sum $\sum_t \psi(t)$ is equal to 1.

In the homology model $M$, each selection $t$ is chosen with probability proportional to $\psi(t)\phi(t)$, where each term is defined in the previous section. The sequences themselves are emitted with probability $P(S|t)$. Probability score of the two sequences under the homology model M:

$$P(S|M) = \sum_t P(S|t) P(t|M) = \sum_t P(S|t) \frac{\psi(t)\phi(t)}{\sum_t \psi(t)\phi(t)} = \frac{\sum_t P(S|t)\psi(t)\phi(t)}{\sum_t \psi(t)\phi(t)} \tag{5}$$

Note that the homology model $M$ differs from null model $nB$ only by the factor of $\phi(t)$ in the probability of selection $t$. This multiplicative weight is defined as $\phi(t) = \prod_m \phi^{n_m(t)} = \phi^{\sum_m n_m(t)}$

(see previous section). The weight $\phi(t)$ rewards selections where $n_m(t)$ is higher (since $\phi > 1$), i.e., where both sequences have a greater number of sites of $m$ selected. The weight is greater for greater values of constant $\phi$, which is set to 5 in our implementation. Setting $\phi = 1$ results in model $M$ being identical to null model $nB$.

Log likelihood ratio (LLR) score for null model $nA$:

$$\text{LLRA}\left(S\right) = \log \frac{P\left(S|M\right)}{P\left(S|nA\right)} \tag{6}$$

Log likelihood ratio for null model $nB$:

$$\text{LLRB}\left(S\right) = \log \frac{P\left(S|M\right)}{P\left(S|nB\right)} \tag{7}$$

# 3 Factorizations

Calculations of the likelihood scores $P(S|nB)$ and $P(S|M)$ in the previous section require summing over all possible site selections $t$, and the number of such selections is exponential in the number of candidate sites. In this section we show how the formulas for likelihood scores may be rewritten for efficient calculation.

## 3.1 An assumption

We assume that candidate sites do not mutually overlap. That is, for any motif $m$ in any sequence $s \in S$, if $t_m^s[k]$ is false, which means that the $k^{th}$ candidate site of motif $m$ in sequence $s$ is not included in selection $t$, then all nucleotide positions in this candidate site are emitted from the background distribution. Given the set of motif matches in any sequence, we greedily select a subset of non-overlapping motif matches to use as candidate sites. This assumption is currently important for efficient calculations of LLR scores, and future work will attempt to relax the assumption.

## 3.2 Equation (4)

Recall that $P\left(S|nB\right) = \dfrac{\sum_t P\left(S|t\right)\psi\left(t\right)}{\sum_t \psi\left(t\right)}$. We next show some simplifications of the numerator and denominator in the formula on the right hand side. These will help rewrite the formula in a way that can be efficiently calculated.

First, consider the denominator:

$$\begin{aligned}\sum_t \psi(t) &= \sum_t \prod_s \left( h_{bg}^s (nw^s) \prod_m \prod_k h_{t_m^s[k]?m:bg}^s (w_m^s[k]) \right) \\ &= \prod_s \sum_{t^s} \left( h_{bg}^s (nw^s) \prod_m \prod_k h_{t_m^s[k]?m:bg}^s (w_m^s[k]) \right) \\ &= \prod_s \left( h_{bg}^s (nw^s) \sum_{t^s} \prod_m \prod_k h_{t_m^s[k]?m:bg}^s (w_m^s[k]) \right) \\ &= \prod_s \left( h_{bg}^s (nw^s) \prod_m \sum_{t_m^s} \prod_k h_{t_m^s[k]?m:bg}^s (w_m^s[k]) \right) \\ &= \prod_s \left( h_{bg}^s (nw^s) \prod_m \prod_k \sum_{t_m^s[k]} h_{t_m^s[k]?m:bg}^s (w_m^s[k]) \right) \\ &= \prod_s \left( h_{bg}^s (nw^s) \prod_m \prod_k \left( h_m^s (w_m^s[k]) + h_{bg}^s (w_m^s[k]) \right) \right)\end{aligned}$$

Note that the outer summation over all site selections $t$ has been pushed inside.

Now consider the summand in the numerator:

$$\begin{aligned}&P(S|t)\,\psi(t) \\ &= \prod_s \left( e_{bg}^s (nw^s) \prod_m \prod_k e_{t_m^s[k]?m:bg}^s (w_m^s[k]) \right) \prod_s \left( h_{bg}^s (nw^s) \prod_m \prod_k h_{t_m^s[k]?m:bg}^s (w_m^s[k]) \right) \\ &= \prod_s \left( e_{bg}^s (nw^s)\, h_{bg}^s (nw^s) \prod_m \prod_k e_{t_m^s[k]?m:bg}^s (w_m^s[k]) \prod_m \prod_k h_{t_m^s[k]?m:bg}^s (w_m^s[k]) \right) \\ &= \prod_s \left( e_{bg}^s (nw^s)\, h_{bg}^s (nw^s) \prod_m \left( \prod_k e_{t_m^s[k]?m:bg}^s (w_m^s[k]) \prod_k h_{t_m^s[k]?m:bg}^s (w_m^s[k]) \right) \right) \\ &= \prod_s \left( e_{bg}^s (nw^s)\, h_{bg}^s (nw^s) \prod_m \prod_k e_{t_m^s[k]?m:bg}^s (w_m^s[k])\, h_{t_m^s[k]?m:bg}^s (w_m^s[k]) \right) \\ &= \prod_s \left( v_{bg}^s (nw^s) \prod_m \prod_k v_{t_m^s[k]?m:bg}^s (w_m^s[k]) \right)\end{aligned}$$

Considering the summation over $t$ in the numerator and "pushing in" of this summation similar to that above gives us:

$$\begin{aligned}\sum_t P(S|t)\,\psi(t) &= \sum_t \prod_s \left( v_{bg}^s (nw^s) \prod_m \prod_k v_{t_m^s[k]?m:bg}^s (w_m^s[k]) \right) \\ &= \cdots = \prod_s \left( v_{bg}^s (nw^s) \prod_m \prod_k \left( v_m^s (w_m^s[k]) + v_{bg}^s (w_m^s[k]) \right) \right)\end{aligned}$$

Therefore:

$$
\begin{aligned}
P\left(S|nB\right) &= \frac{\sum_t P\left(S|t\right)\psi\left(t\right)}{\sum_t \psi\left(t\right)} \\
&= \frac{\prod_s \left(v_{bg}^s\left(nw^s\right)\prod_m\prod_k\left(v_m^s\left(w_m^s\left[k\right]\right)+v_{bg}^s\left(w_m^s\left[k\right]\right)\right)\right)}{\prod_s \left(h_{bg}^s\left(nw^s\right)\prod_m\prod_k\left(h_m^s\left(w_m^s\left[k\right]\right)+h_{bg}^s\left(w_m^s\left[k\right]\right)\right)\right)}
\end{aligned} \tag{8}
$$

In this simplified formula both the numerator and the denominator can be computed by computing a single summation (of two whole scores or two shell scores) for each candidate site and then multiplying across all candidate sites over all motifs and both sequences. Thus the time complexity is linear in the total number of candidate sites.

### 3.3 Equation (5)

We begin by a simple rewriting of $\psi(t)$:

$$
\begin{aligned}
\psi\left(t\right) &= \prod_s\left(h_{bg}^s\left(nw^s\right)\prod_m\prod_k h^s_{t_m^s[k]?m:bg}\left(w_m^s\left[k\right]\right)\right) \\
&= \left(\prod_s h_{bg}^s\left(nw^s\right)\right)\left(\prod_s\prod_m\prod_k h^s_{t_m^s[k]?m:bg}\left(w_m^s\left[k\right]\right)\right) \\
&= \left(\prod_s h_{bg}^s\left(nw^s\right)\right)\left(\prod_m\prod_s\prod_k h^s_{t_m^s[k]?m:bg}\left(w_m^s\left[k\right]\right)\right)
\end{aligned}
$$

Also:

$$
\begin{aligned}
P\left(S|t\right)\psi\left(t\right) &= \prod_s\left(v_{bg}^s\left(nw^s\right)\prod_m\prod_k v^s_{t_m^s[k]?m:bg}\left(w_m^s\left[k\right]\right)\right) \\
&= \cdots = \left(\prod_s v_{bg}^s\left(nw^s\right)\right)\left(\prod_m\prod_s\prod_k v^s_{t_m^s[k]?m:bg}\left(w_m^s\left[k\right]\right)\right)
\end{aligned}
$$

Recall that $P(S|M) = \dfrac{\sum_t P\left(S|t\right)\psi\left(t\right)\phi\left(t\right)}{\sum_t \psi\left(t\right)\phi\left(t\right)}$, and that $\phi(t) = \prod_m \phi_m(t)$. We define a new notation $\phi(t_m) \equiv \phi_m(t)$ for use below.

We first consider the sum in the denominator:

$$\sum_t \psi(t)\,\phi(t) = \sum_t \left(\prod_s h^s_{bg}(nw^s)\right)\left(\prod_m \prod_s \prod_k h^s_{t^s_m[k]?m:bg}(w^s_m[k])\right)\left(\prod_m \phi(t_m)\right)$$
$$= \sum_t \left(\prod_s h^s_{bg}(nw^s)\right)\left(\prod_m \left(\phi(t_m)\prod_s \prod_k h^s_{t^s_m[k]?m:bg}(w^s_m[k])\right)\right)$$
$$= \left(\prod_s h^s_{bg}(nw^s)\right)\left(\sum_t \prod_m \left(\phi(t_m)\prod_s \prod_k h^s_{t^s_m[k]?m:bg}(w^s_m[k])\right)\right)$$
$$= \left(\prod_s h^s_{bg}(nw^s)\right)\left(\prod_m \sum_{t_m} \left(\phi(t_m)\prod_s \prod_k h^s_{t^s_m[k]?m:bg}(w^s_m[k])\right)\right)$$

Next considering the numerator, the summation over $t$ can be "pushed in" in a similar fashion:

$$\sum_t P(S|t)\,\psi(t)\,\phi(t) = \sum_t \left(\prod_s v^s_{bg}(nw^s)\prod_m \prod_s \prod_k v^s_{t^s_m[k]?m:bg}(w^s_m[k])\prod_m \phi(t_m)\right)$$
$$= \cdots = \left(\prod_s v^s_{bg}(nw^s)\right)\left(\prod_m \sum_{t_m} \left(\phi(t_m)\prod_s \prod_k v^s_{t^s_m[k]?m:bg}(w^s_m[k])\right)\right)$$

Therefore:

$$P(S|M) = \frac{\sum_t P(S|t)\,\psi(t)\,\phi(t)}{\sum_t \psi(t)\,\phi(t)}$$
$$= \frac{\left(\prod_s v^s_{bg}(nw^s)\right)\left(\prod_m \sum_{t_m}\left(\phi(t_m)\prod_s \prod_k v^s_{t^s_m[k]?m:bg}(w^s_m[k])\right)\right)}{\left(\prod_s h^s_{bg}(nw^s)\right)\left(\prod_m \sum_{t_m}\left(\phi(t_m)\prod_s \prod_k h^s_{t^s_m[k]?m:bg}(w^s_m[k])\right)\right)} \quad (9)$$

To calculate either the numerator or denominator, we enumerate all possibilities of $t_m$ and compute the sum $\left(\phi(t_m)\prod_s \prod_k \cdots\right)$. This has time complexity exponential in the number of candidate sites of motif $m$. The summation over $t_m$ is calculated separately for each $m$ and a product of these summations is taken. Thus, the overall time complexity is exponential in the number of candidate sites for the most frequent motif but not exponential in the total number of candidate sites over all motifs.
