# Supplementary material for: Evidence for Deep Regulatory Similarities in Early Developmental Programs across Highly Diverged Insects
Source: Genome Biol Evol. 2014 Aug 29;6(9):2301–20. doi: 10.1093/gbe/evu184 (PMC4217690; doi:10.1093/gbe/evu184)
Supplement: Supplementary Data [file supp_evu184_evu184-suppl_data.zip › Supplementary_Tables&Figures.pdf]

Supplementary Information

Evidence for deep regulatory similarities in early developmental programs across highly diverged insects

Majid Kazemian[1,2,*], Kushal Suryamohan[3,5,*], Jia-yu Chen[1,*], Yinan Zhang[1], Md. Abul Hassan Samee[1], Marc S. Halfon[3,4,5,6,!] and Saurabh Sinha[1,7,!]

[1]Department of Computer Science and [7]Institute of Genomic Biology, University of Illinois at Urbana-Champaign, Urbana, IL 61801, USA.
[2]Laboratory of Molecular Immunology, National Heart Lung and Blood Institute, National Institutes of Health, Bethesda, MD 20824, USA.
[3]Department of Biochemistry and [4]Department of Biological Sciences University at Buffalo-State University of New York, Buffalo, NY 14203, USA.
[5]NY State Center of Excellence in Bioinformatics and Life Sciences, Buffalo, NY 14203, USA.
[6]Molecular and Cellular Biology Department and Program in Cancer Genetics, Roswell Park Cancer Institute, Buffalo, NY 14263, USA.

*equal contribution
!co-corresponding authors

MSH:
University at Buffalo-State University of New York
701 Ellicott St.
Buffalo, NY 14203
(716) 829-3126
mshalfon@buffalo.edu

SS:
University of Illinois, Urbana-Champaign
2122 Siebel Center, 201 N. Goodwin Ave,
Urbana, IL 61801
(217) 333-3233
sinhas@illinois.edu

Table S1. Similarity in developmental gene expression among *A. mel, N. vit, T. cas,* and *An. Gam.*

Table S2. Evaluation of cross species supervised CRM prediction. Evaluation p-value and the overlap size of the enrichment test over 5 arthropod species from 3 different statistical models. The first and second column of the "Evaluation_pvalue" worksheet show the names of 36 data sets (i.e., training sets of *Drosophila* CRMs defined by spatio-temporal domain of regulatory function) and their relevant expression gene sets, respectively. The next 15 columns report the evaluation p-value (Hypergeometric test) of three statistical models in *Drosophila* and four other insect species. The best evaluation p-values among different statistical models are colored orange. The "overlap_size" worksheet is laid out similar to the "Evaluation_pvalue" worksheet except that it includes the raw information about the enrichment tests: the "Universe size", the "Gene Set size" (expression gene set), the "Sample Size" (top genes near predicted CRMs), and the overlap size (intersection between the two sets) for each statistical model and each species.

Table S3. Evaluation p-values of motif-based approach. Evaluation p-value of the enrichment test for 4 arthropod species using Stubb-MDB (1). Stubb-MDB was run with the motifs from *Drosophila* listed in the last column. The Stubb-MDB was found to have equal or better results when compared to another motif-based approach (1). Moreover, it is an in-house program, so we have more expertise in tuning its parameters for the best results. Therefore, we only employed Stubb-MDB for this exercise.

Training_CRM_Set	Expresion_GeneSet	#CRMs	Drosophila	Anopheles	Nasonia	Tribolium	motifs
ap	blastoderm_ap_2004_FB	55	4E-07	0.022	0.2	0.017	BCD, CAD, CIC, FKH, GT, HB, HKB, KNI, KR, TLL, VFL
dv	allDV.genes	19	9E-11	1E-04	0.2	6E-05	CIC, BRK, DL, ESPL, IND, SNA, TWI, VND, ZEN, VFL

Table S4. Candidate CRMs in four non-*Drosophila* species. The “Candidate enhancers” worksheet lists all putative CRMs predicted using our supervised CRM prediction scheme. The first column shows the name of the species in which the CRM is predicted. The second and third columns report the method (statistical model) and the training data set used for predicting the CRM. The next four columns show the coordinates, the two neighboring genes, the global rank and the local rank of the predicted CRM, respectively. The final column denotes if the CRM was tested in vivo (see Table S3). Note that a CRM can be predicted by several datasets and several methods; therefore this list contains redundant candidate CRMs.

Table S5. Evaluation of three statistical models on known CRMs in *A. gam (Anopheles)*, *A. mel (Apis)*, and *T. cas (Tribolium)*. For each CRM and each of three statistical models, the global (genome-wide) rank of the CRM's score as per the statistical model, using the training set mentioned in column "Related CRM set," is shown. The best rank in each row is highlighted, if it is < 4000 (i.e. ~0.5% of all segments in the target genome). The given evaluation p-value is the best from among the three statistical models.

					Global rank		
	**CRM**	**Species**	**Related CRM set**	**Evaluation P-value**	**msHexMCD**	**msIMM**	**PACRC**
**1**	**brk**	*Anopheles*	dv	2E-08	94	24	99
**2**	**sim**	*Anopheles*	dv	2E-08	>4000	3412	2577
**3**	**sog_1**	*Anopheles*	dv	2E-08	275	>4000	60
**4**	**sog_2**	*Anopheles*	dv	2E-08	>4000	>4000	>4000
**5**	**twi**	*Anopheles*	mesectoderm	1E-05	1841	>4000	>4000
**6**	**vnd**	*Anopheles*	dv	2E-08	>4000	>4000	126
**7**	**vnd_intronic**	*Anopheles*	dv_earlymesoderm	2E-04	>4000	>4000	2394
**8**	**sim_1**	*Apis*	dv	2E-10	2139	2712	1226
**9**	**sim_2**	*Apis*	mesectoderm	7E-08	>4000	3699	>4000
**10**	**ase**	*Tribolium*	cns	2E-13	>4000	1321	>4000
**11**	**cact**	*Tribolium*	blastoderm	1E-12	220	1391	260
**12**	**h_0.8**	*Tribolium*	blastoderm	1E-12	>4000	>4000	2193
**13**	**h_1.9**	*Tribolium*	blastoderm	1E-12	1270	>4000	2193
**14**	**hb**	*Tribolium*	ap	5E-08	2415	2225	312
**15**	**sim**	*Tribolium*	dv	4E-08	272	37	>4000
**16**	**sog**	*Tribolium*	blastoderm	1E-12	>4000	1323	>4000

Table S6. Experimentally validated CRMs in diverged species. The 24 candidate CRMs tested in vivo are provided. Note that when individual high scoring segments (Table S5) overlap, the segments are merged into a single candidate CRM for validation. Additional details can be found in the notes at the end of the Table.

Table S7. Accuracy numbers from experimental validation, when a predicted CRM is required to meet the prediction criteria from two statistical models rather than one. Precision = 83%, Recall = 63%, F1 score = 71%, specificity = 75%.

Predicted / Confirmed	Yes	No	Total
**Yes**	10	6	16
**No**	2	6	8
**Total**	12	12	24

Table S8: Primer sequences used for experimental validation.

Candidate CRM	Primer	Sequence
Apis mellifera		
Amel_lab_1	lah1f	GTTTGGGAAACTCGTTCGTCGATAC
	lah1r	CACGCGTGTGTACACAGAAGAA
Tcas_wg_1	wah2f	AGGAGCTCCTCGGGGTCC
	wah2f2	CGGTATTGTAATTGTTCGGAATTTTGT
Tcas_wg_2	wgamel-neg2f	AAGCAAATCATAAACGTCTCG
	wgamel-neg2r	TCCCCGACCAATTCCGGAGAA
Amel_h_1	hai2f	ATAATATATCTACGGTCACGCGACG
	hai2r	TTCTTCACGAGATTGCCAGACTGT
Amel_oc_1	oai3f	GCATTAAGGATCCACGCAATGT
	oai3r	CGATTTGCCGTCCCTTTCG
Amel_ttk_1	ttk_Amel-rnd33f	TACGAGAGTTTGTTGCTGCG
	ttk_Amel-rnd33r	AAAATATTCGCCAGCGTGAC
Amel_ttk_2	ttkamel-neg6f	TACGTTGTACGCGTTTCGAG
	ttkamel-neg6r	CACGCAATACGTGTCGATCT
Amel_Sox21b_1	sox21b-rnd31f	GTTCACCGAAATTGAAACGG
	sox21b-rnd31r	TCAAGATGCCGATGTATGGA
Nasonia vitripennis		
Nvit_h_m8	hni8f	GTTCCACAAAATATCCTTTGCCGA
	hni8r	TTGCCGCCGACTCTAGGTAG
Nvit_h_m1	h_Nvit-rnd34f	TTATGCTCTGTTTTGAGCGG
	h_Nvit-rnd34r	TCCGATGTACACAAACACGG
Nvit_h_m4	h_Nvit-50f	GCGTCCCGTTGAAACTTTTT
	h_Nvit-50r	AGCTACGGTGGTTGCTTTTT
Nvit_h_2	hnvit-neg5f	TGCACAGCTGGAAATTGTCT
	hnvit-neg5r	AAGATCGCGAACGACTGATT
Nvit_neur_1	neur_Nvit-73f	ACAAACGCTGTGAACAATCG
	neur_Nvit-73r	CGAGAGGAGAGGAGGACAAA
Nvit_neur_2	neurnvit-neg3f	CCGCAAGATTGTGGTAGCTT
	neurnvit-neg3r	AAAGCGAGGCGCTGTACTTA
Tribolium castaneum		
Tcas_lab_1	lth1f	GCAAATTGTTTTTGACGAAATGACAC
	lth1r	ATATCTCGACAGTTCCTAATTAAGGAA
T_cas_wg_1	wth1f	ATGACTTGTTCAAGTGTTGTTAGTCG
	wth1r	CCGATGTGGCGTTTTTCAGGTA
Tcas_wg_2	wgtcas-neg1f	ATTTACGCTTTCTGAACTCGAT
	wgtcas-neg1r	CAGTTGTGTATTTCCCATGGCGT
Tcas_D_1	D_Tcas-14f	GGCGAAAGCGTTTGAAAATA
	D_Tcas-14r	GGCAACAGTTCTCCGATTGT
Tcas_D_2	Dtcas-neg4f	ATTTTGGTTCAAGGGTGCAA
	Dtcas-neg4r	CGAAGCCCTGCAAGTTTTTA

Tcas_pb_1	pb_Tcas-rnd32f	TCCGAGGTTAGTTAGGTCCG
	pb_Tcas-rnd32r	AAAAAGCTGAGTGCGTTCG
Tcas_psq_1	psq_Tcas-67f	AAAAGCCCCAATTAAAACCG
	psq_Tcas-67r	TCAATTTTCGCAACTCGTTT
Anopheles gambiae		
Agam_gt_1	gt_Agam-45f	GTGTGTCCTCACCGAAGGTC
	gt_Agam-45r	GAGAACCACTTCTCCCACGA
Agam_h_1	h_Agam_rnd41f	AAGTAACTCTCCCAGCACCG
	h_Agam_rnd41r	AACTGTTGGCAAAAAGGCTG
Agam_sog_1	sog_Agam-98f	AGCGCAGGGTGTTCTGTT
	sog_Agam-98r	AAGCAGCGCAGCACCCGCAA

Table S9: Tukey's multiple comparison test results for BLAST hits.

Figure S1. Alignment of non-coding regions. Left-hand panels show dotplot alignments of non-coding regions from the *D. mel D/Sox-21b* (A), *en* (B), *gt* (C), *h* (D), *sim* (E), *sog* (F), *wg* (G), and *twi* (H) loci aligned to orthologous loci in a more distant Drosophilid (left, *D. virilis* or *D. mojavensis*, ~40-60 My, except *twi*, which is *D. willistoni*), one of the other insects in our study (middle, *An. gam, T. cas, or A. mel*), and to a randomized version of the other-insect sequence (right). The right-hand panels show boxplots of the distribution of the top BLAST bit scores for the alignments.

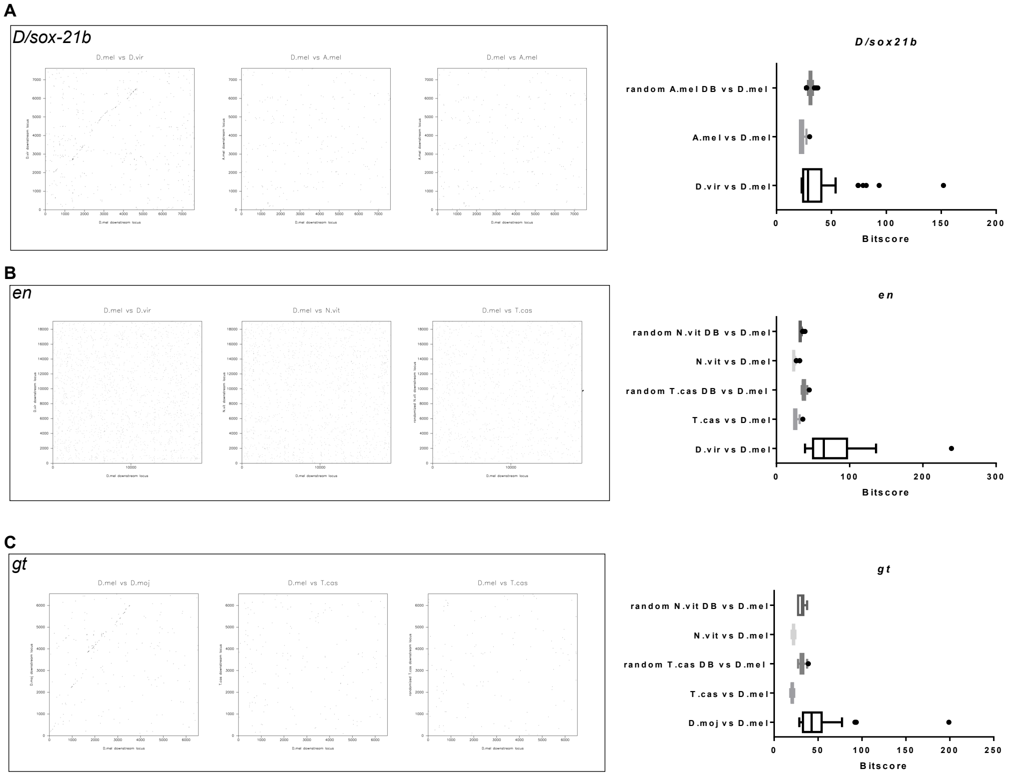

D

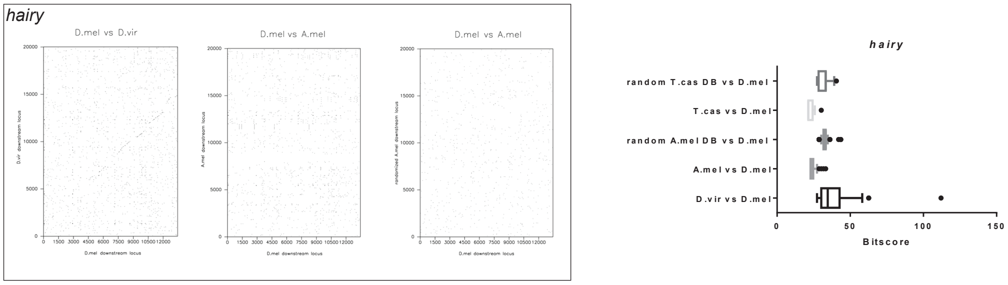
hairy
D.mel vs D.vir
D.mel vs A.mel
D.mel vs A.mel
hairy
random T.cas DB vs D.mel
T.cas vs D.mel
random A.mel DB vs D.mel
A.mel vs D.mel
D.vir vs D.mel
0
50
100
150
Bitscore

E

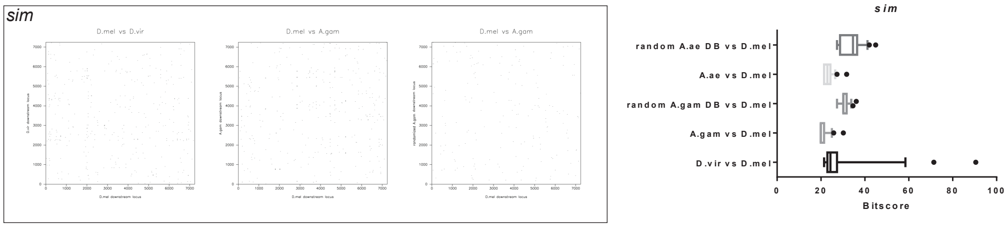
sim
D.mel vs D.vir
D.mel vs A.gam
D.mel vs A.gam
sim
random A.ae DB vs D.mel
A.ae vs D.mel
random A.gam DB vs D.mel
A.gam vs D.mel
D.vir vs D.mel
0
20
40
60
80
100
Bitscore

F

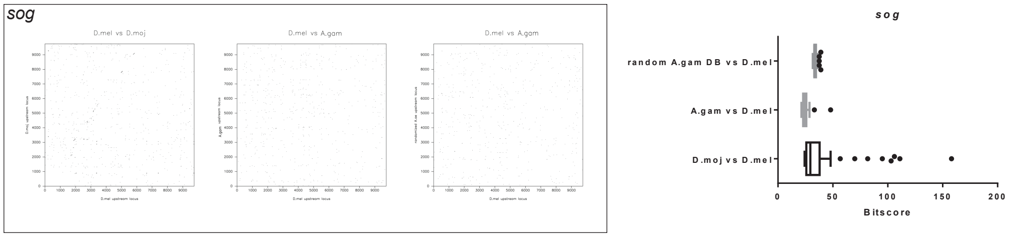
sog
D.mel vs D.moj
D.mel vs A.gam
D.mel vs A.gam
sog
random A.gam DB vs D.mel
A.gam vs D.mel
D.moj vs D.mel
0
50
100
150
200
Bitscore

G

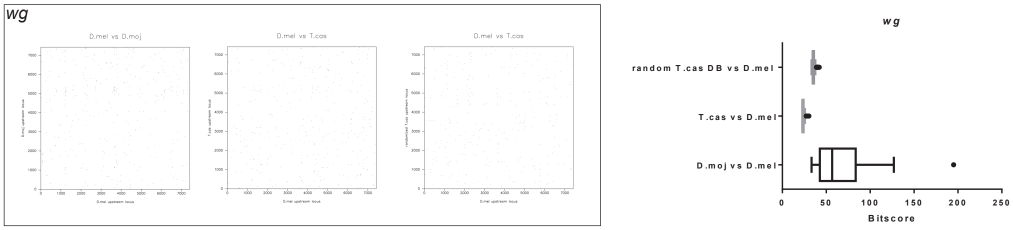
wg
D.mel vs D.moj
D.mel vs T.cas
D.mel vs T.cas
wg
random T.cas DB vs D.mel
T.cas vs D.mel
D.moj vs D.mel
0
50
100
150
200
250
Bitscore

H

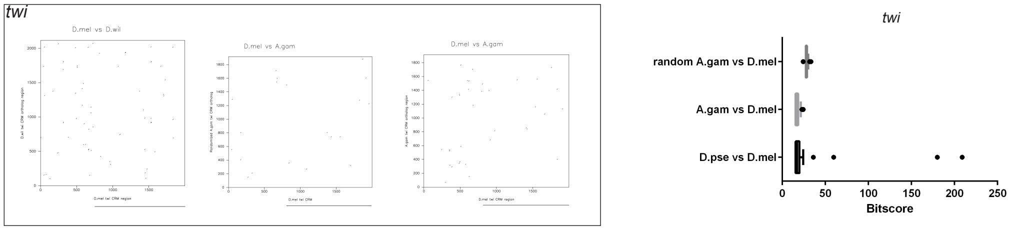
twi
D.mel vs D.wil
D.mel vs A.gam
D.mel vs A.gam
twi
random A.gam vs D.mel
A.gam vs D.mel
D.pse vs D.mel
0
50
100
150
200
250
Bitscore

Figure S2. Comparison of evaluation p-values between different statistical models of supervised CRM prediction. Each point represents a data set and each axis represents –log (base 10) of the evaluation p-value corresponding to a statistical model.

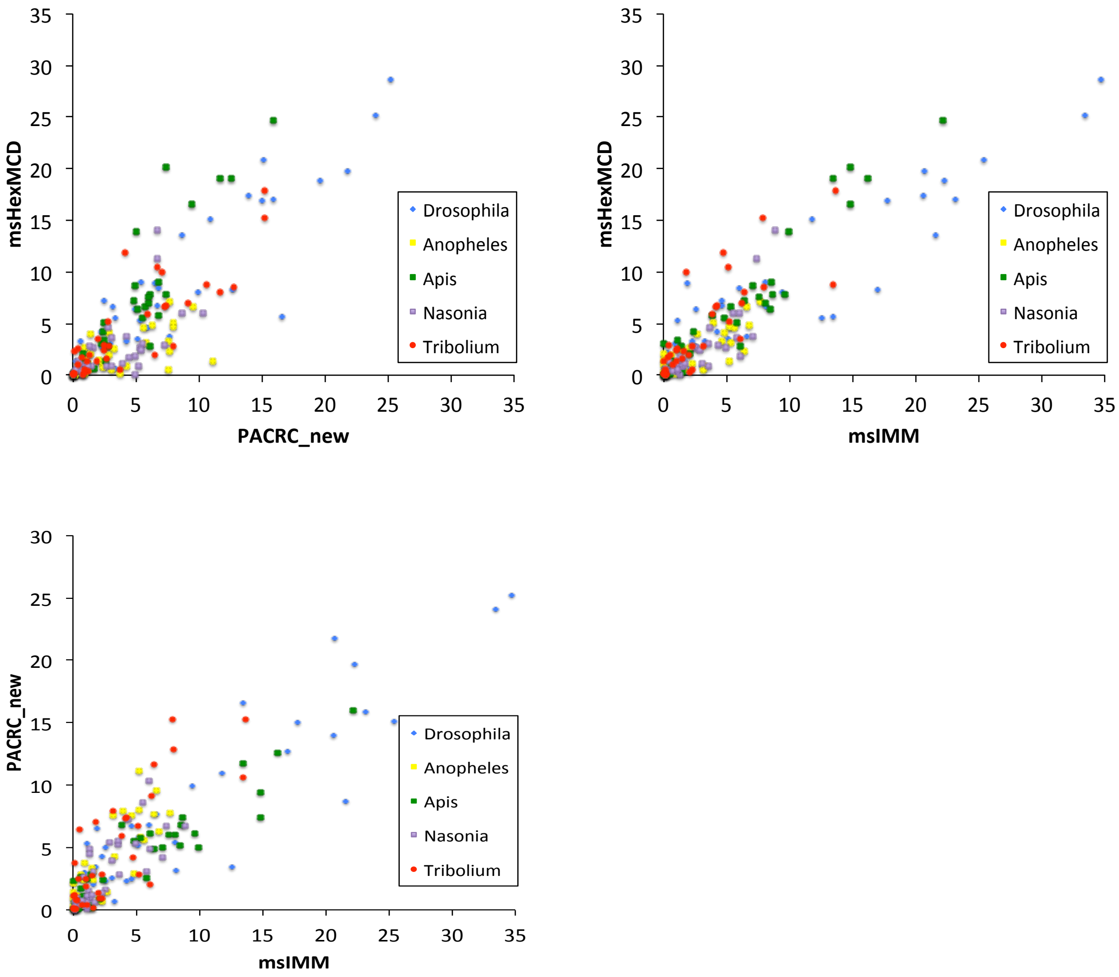

Figure S3: Experimentally validated CRMs. See also Figure 5a. Predicted CRM sequences were used to drive GFP reporter gene expression in transgenic *Drosophila* embryos. GFP expression was visualized using antibodies to GFP in all panels except for E, which uses in situ hybridization to mRNA. All embryos are shown with anterior to the left. Panels A, D, H, I, J, K, and M are dorsal views; F, G, and L are ventral views; B, C, E, N, and O are lateral views with dorsal to the top. (A) Late expression of the GFP reporter gene for the same *A. gam. sog* CRM (*Agam_sog_1*) depicted in Figure 5a.I. Expression is observed in the brain (arrowhead) as well as a segmentally repeated pattern in the ectoderm. (B) Additional view of the *N. vit. neur* CRM (*Nvit_neur_1*; see Figure 5a.B) showing reporter gene expression in the peripheral nervous system (arrows). (C) A predicted CRM for the *A. mellifera Sox21b* gene drives GFP expression in a segmentally repeated pattern similar to that expected for *Drosophila Sox21b*. (D, E) A predicted CRM for the *hairy* gene from *A. mel.* shows reporter gene expression in the brain and visceral mesoderm, as well as in the posterior spiracles. This expression may represent perdurance of the GFP protein from earlier expression in these tissues, as reported by (2, 3). (F) The predicted CRM *Amel_ttk_1*, shown in Figure 5a.G and 2a.H additionally regulates expression in the ventral midline, consistent with endogenous Ttk expression. (G) A "false negative" prediction in the *A. mel. ttk* locus gives strong expression in a subset of somatic muscles. (H) Endogenous *labial* gene expression in a stage 9 embryo; compare with the embryo in (I) where a predicted CRM for the *labial* gene in *A. mel.* drives expression of the reporter gene in a similar pattern. (J, K)) A predicted CRM for the *labial* gene in *T. cas.* drives weak expression in the gut as well as in segmentally-repeated ectodermal clusters. As these tissues are not expected to be strong sites of *lab* expression, we classify this CRM as the only of our "true positive" predicted CRMs that does not clearly recapitulate the expression of its most likely associated gene. (L) False negative prediction from the *T. cas. D* locus. Although *Drosophila D* is expressed in a segmentally repeated pattern in the nerve cord, it does not express in broad ecotdermal stripes as seen here. (M) Expression regulated by a predicted CRM for *gt* in *A. gam.* is observed in a cluster of cells in the brain (arrowhead) similar to that seen with endogenous *gt* in *Drosophila*. (N) Unlike the false negative predicted CRM *Amel_wg_2* described in Figure 5a.J, which drives expression in a broad, non-Wg-like pattern, this true-positive predicted *A. mel. wg* CRM drives ventral expression along the Wg stripe in a pattern highly similar that observed for the *Tcas_wg_2* CRM shown in (O), which is a "false negative" prediction. In both, significant overlap with ventral Wg expression can be observed. Green, GFP; magenta, Wg.

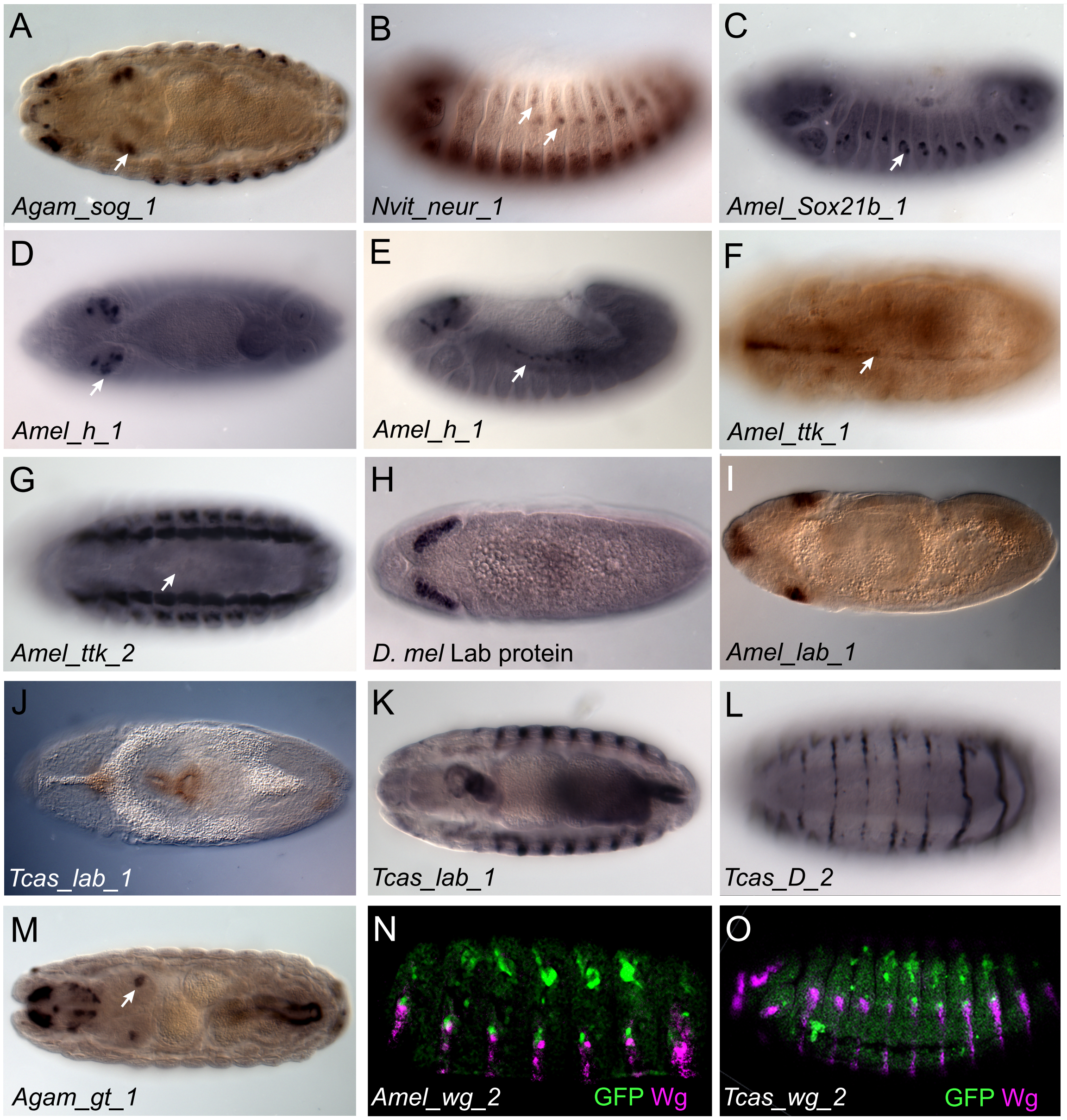
A
Agam_sog_1
B
Nvit_neur_1
C
Amel_Sox21b_1
D
Amel_h_1
E
Amel_h_1
F
Amel_ttk_1
G
Amel_ttk_2
H
D. mel Lab protein
I
Amel_lab_1
J
Tcas_lab_1
K
Tcas_lab_1
L
Tcas_D_2
M
Agam_gt_1
N
Amel_wg_2
GFP Wg
O
Tcas_wg_2
GFP Wg

Figure S4: TF binding site motifs in functionally related CRM pairs.

(a) *hairy* stripe CRMs from *Drosophila* and *Nasonia*. The *Nvit_h_m8* CRM (this study, Figure 5E) is pictured at the top, followed by aligned sequences of the *Dmel_h_6+2* CRM (4) and the orthologous sequences from *D. sechelia, D. yakuba, D. pseudoobscura, D. willistoni,* and *D. virilis.* Horizontal green bars indicate the extent of the sequences tested in vivo. Dashed lines indicate motif similarities between the *D. mel* and *N. vit* CRMs. Solid black lines indicate motifs conserved in orthologous sequences within the Drosophilids; wedges indicate that a cluster of motifs is maintained. For clarity, only a subset of conserved motifs are marked.

(b) Motif alignment of the *D. mel "brk_shadow" brk* CRM and the orthologous sequence from five other Drosophilids with the *An. gam brk* CRM defined by Cande et al. (5). The horizontal green bar indicates the extent of the annotated *D. mel* CRM; the entire displayed *An. gam* region was tested for activity. Dashed black lines link similar motifs between *Drosophila* and *Anopheles.* Note the conserved arrangement of motifs as evidenced by a general lack of crossing of the lines; an exception for a Twi binding motif is shown by a dotted line. A heavy black arrow indicates a *Drosophila*-specific CAPICUA (CIC) motif.

(c) Motif alignment of the *Drosophila sim_mesectoderm* CRM from *D. mel* and three other species, along with alignments of the *An. gam sim* CRM from Markstein et al. (6) and the *A. mel sim* CRM from Zinzen et al. (7). The horizontal green bar indicates the extent of the annotated *D. mel* CRM. Blue bars highlight the similar "core" motif arrangement of sites for DORSAL, ZELDA, and SNAIL. Jagged black lines in the *Anopheles* alignments denote a gap introduced for purposes of clarifying the alignment. Dashed black lines connect similar motifs between *Drosophila* and either mosquito or bee alignments within the core region. Note the conserved arrangement of motifs as evidenced by lack of crossing of the dashed lines, particularly between flies and bees. Solid black lines link conserved motifs within a genus.

(d) Motif alignment of the *Drosophila Dichaete D_Dfsh_O-E* CRM from *D. mel* and ortholgoous regions from five other species, along with the *T. cas Tcas_D_1 CRM* (this study). Horizontal green bars denote the tested CRM sequences. Dashed black lines show motif similarity between *D. mel* and *T. cas*, while solid black lines show motif conservation within the *Drosophila*. Wedges indicate that multiple motifs align. Note the general similarity in motif composition and arrangement. A major difference is the sharp reduction in Bcd/Otd binding sites in *T. cas* compared to *D. mel*; this may help to account for the absence of early blastoderm-stage expression driven by the *T. cas* CRM despite the close correspondence of the later embryonic patterns.

(e) Alignment of motifs for the *Drosophila wg_Δwg* CRM in *D. mel* plus five other species (bottom) along with the *T. cas Tcas_wg_1* CRM (this study, middle) and *A. mel Amel_wg_1* CRM (this study, top) and three orthologous bee sequences.

Horizontal green bars indicate the tested CRM sequences. No clear alignment of motifs is observed among the three CRMs, although there is overall similarity in motif composition. Note that although the *wg_Δwg* CRM drives mid-to-late embryonic gene expression, the motifs used in this alignment are drawn primarily from early embryonic anterior-posterior patterning. It is possible, therefore, that a more closely related CRM-to-CRM motif mapping would be observed, similar to those seen for the CRMs in panels (a)-(d), if a different, more appropriate set of motifs were to be used.

Species abbreviations: Bees: *Lalb, Lasioglossum albipes; Hlab, Habropoda laboriosa; Bimp, Bombus impatiens; Aflo, Apis florea; Amel, Apis mellifera.* Beetles: *Tcas, Tribolium castaneum.* Flies*: Dmel, Drosophila melanogaster; Dsec, D. sechellia; Dyak, D. yakuba; Dpse, D. pseudoobscura; Dana, D. ananassae; Dwil, D. willistoni; Dvir, D. virilis.* Mosquitoes: *Agam, Anopheles gambiae; Aara, An. arabiensis; Aepi, An. eprioticus; Aqua, An. quadriannulatus; Amer, An. merus; Amelas, An. melas.* Wasps: *Nvit, Nasonia vitripennis*.

Figure S4a: *h_stripe_6+2* CRM, *Drosophila* and *Nasonia*

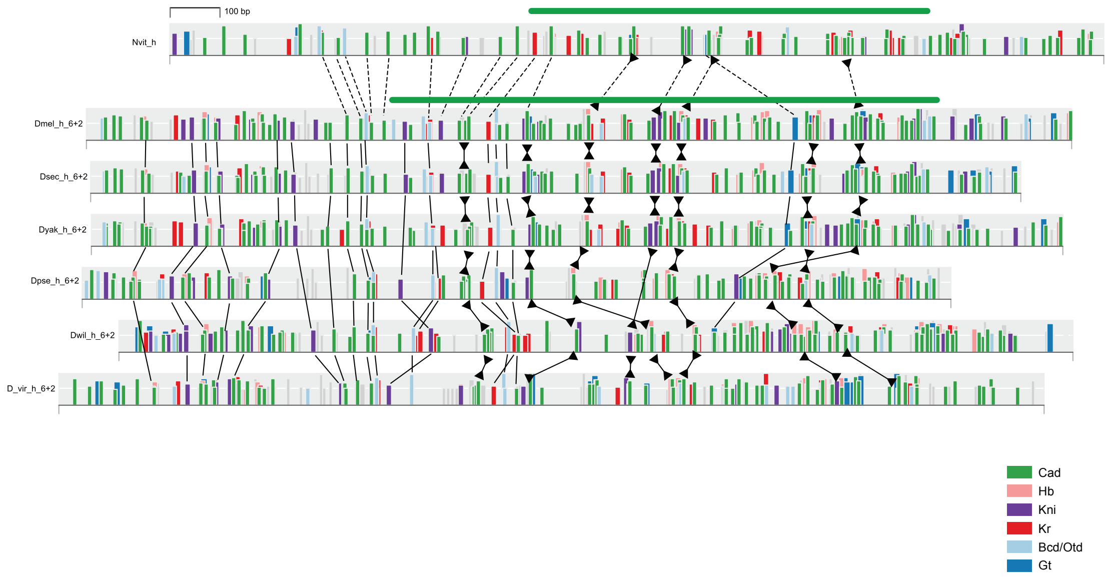

Figure S4b: *brk_shadow* CRM, Drosophila and *Anopheles*

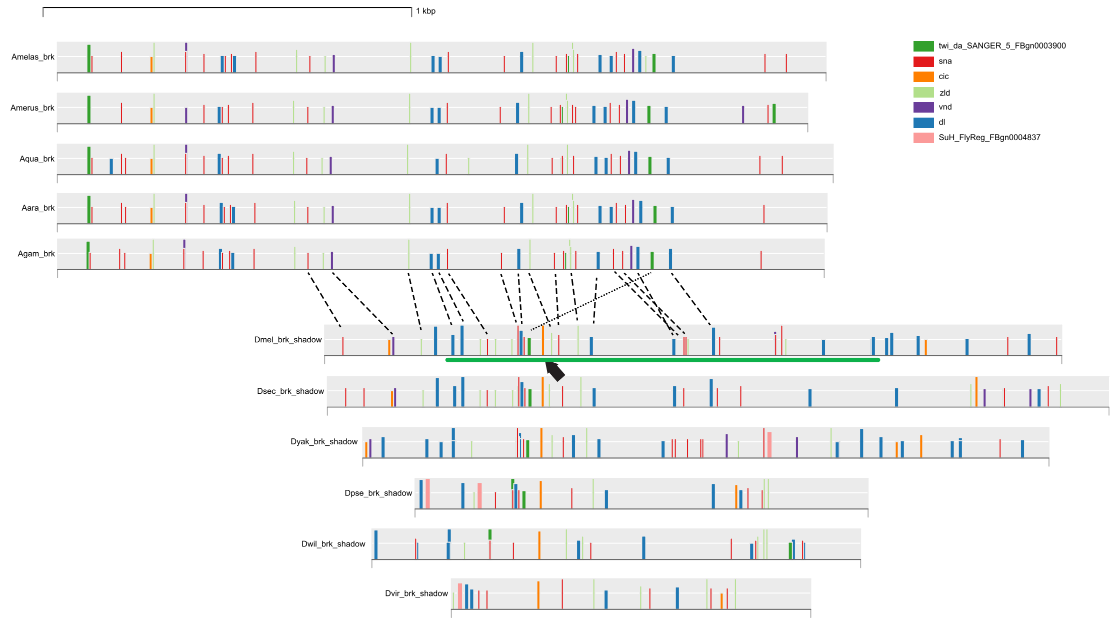

Figure S4c: *sim_mesectoderm* CRM, *Drosophila, Anopheles*, and bees

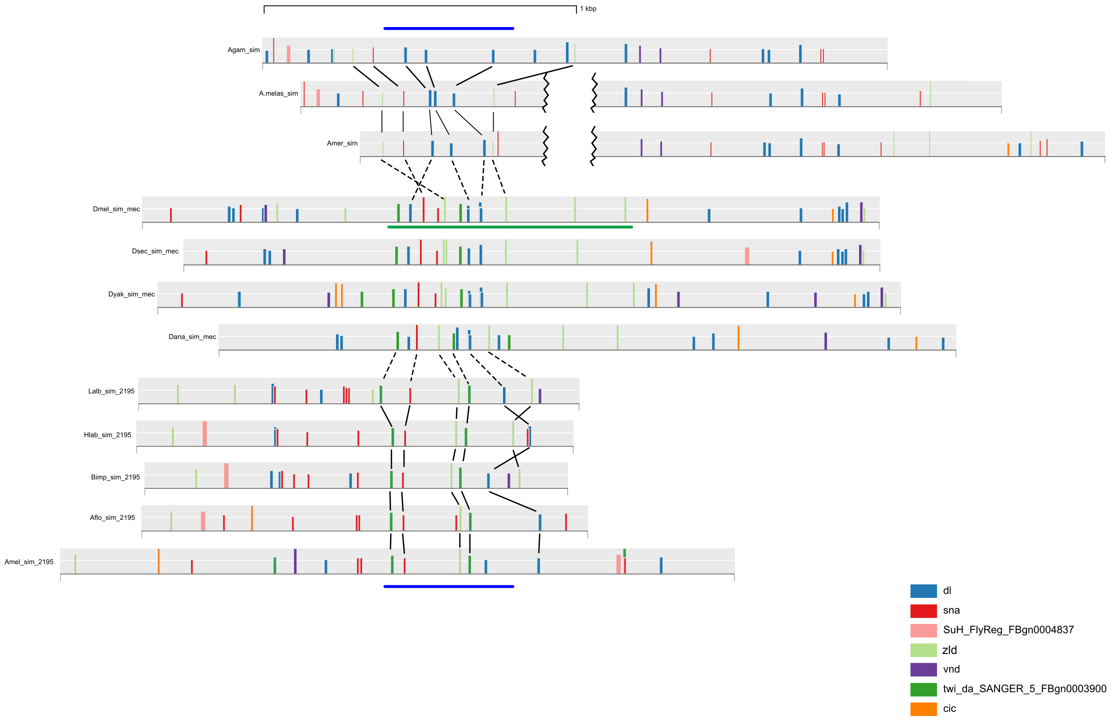

Figure S4d: *D_Dfsh_O_E CRM, Drosophila* and *Tribolium*

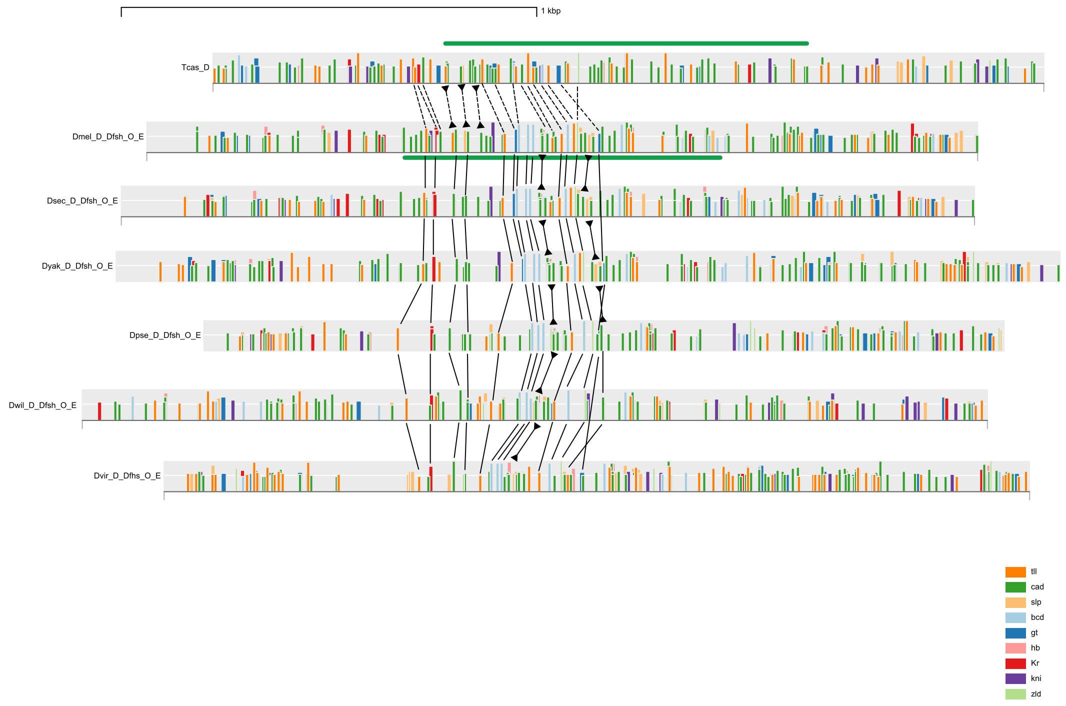

Figure S4e:
wg_∆wg CRM, *Drosophila, T. cas*, and bees

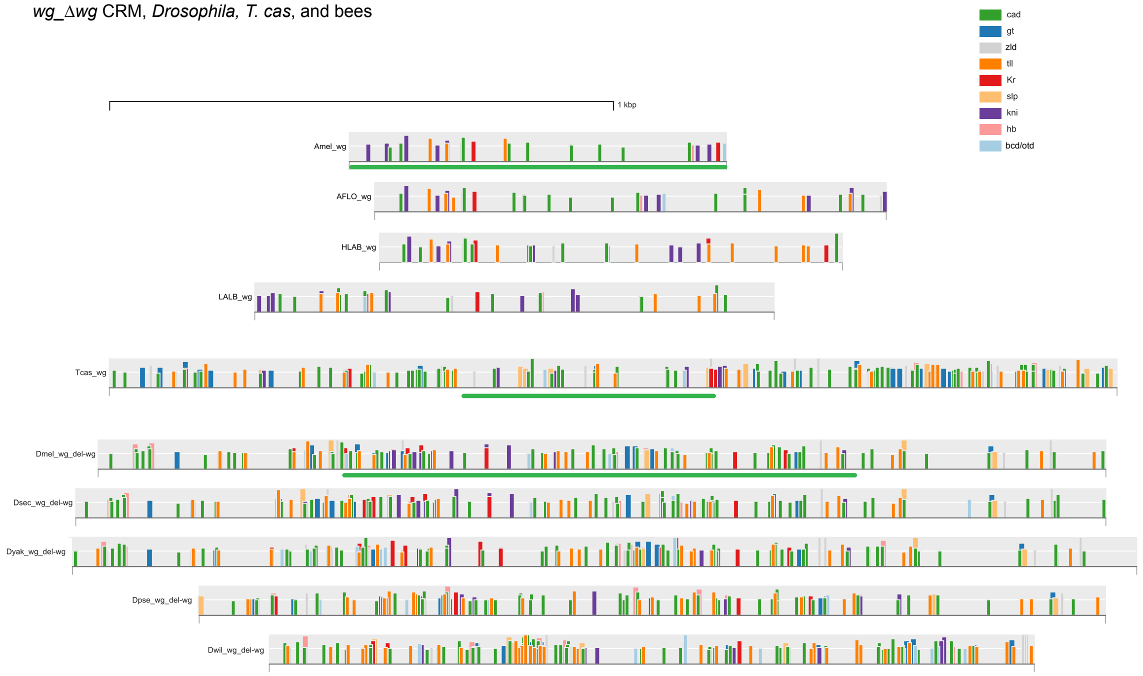

Supplementary Note 1: Known CRMs in other insects. Our count of CRMs described for these four insects includes only what we consider as "well-defined" CRMs, that is, discrete distal DNA sequences demonstrated to regulate gene expression. We do not include promoter fusions extending from the transcription start site 5' to the gene for an arbitrary distance as such constructs, although undeniably useful for experimental purposes, do not clearly demarcate the relevant regulatory sequences. Even including such sequences, the total number remains small.

Supplementary Note 2: Alignment of non-*Drosophila* enhancers using BLAST. To further confirm that our newly-discovered CRMs did not align to the orthologous *Drosophila* loci, we used a sensitive BLASTn assay (word size = 7) to search each CRM against the *D. melanogaster* genome. Neither the previously known nor newly discovered CRMs had even a single BLAST hit within 70 kb of the gene (median distance = 871 kb), with the exception of the newly-discovered *Tcas_D_1* CRM, which mapped just within the *Dichaete* locus (i.e., within its intergenic region) at a distance of 42.5 kb from the gene. Furthermore, none of the CRMs had multiple clustered BLAST hits.

By comparison, we also tested CRMs for the *Themira putris even skipped* gene. Although the original report of the discovery of these CRMs (8) considered them unalignable with the homologous *D. mel* CRM sequences, subsequent more sensitive analysis showed that reasonable alignments could in fact be made (9). We used the *T. putris eve stripe2, MHE*, and *stripe4/6* CRMs in our BLAST assay as a means of gauging its sensitivity. All three CRMs had BLAST hits within 10 kb of the *D. mel eve* gene and in close proximity to the known *D. mel* CRMs; both stripe enhancers had multiple clustered hits.

Supplementary Note 3: Motif Cosine Similarity (MoCS) score. Here, two sequences are compared for their similarity of binding site composition. A set of relevant motifs is given as input. First, each sequence is scored for the presence of each motif by (a) counting the number of matches of the motif with LLR p-value score (obtained from PATSER program (10)) below a threshold, (b) ranking this match count in comparison to an empirical distribution formed from analogous counts in randomly selected non-coding segments and (c) using the negative log of the resulting rank (expressed as a fraction) as the score of the motif. Then, each sequence is represented by a vector of motif scores, with each dimension of the vector corresponding to the score of a motif. The two vectors (say u and v) are then compared using the cosine similarity score, a common measure used in information retrieval (11):

$$\cos(\mathrm{u},\mathrm{v}) = \frac{\sum \mathrm{u_i v_i}}{\sqrt{\sum \mathrm{u_i^2} \sum \mathrm{v_i^2}}}$$

We used a collection of ~250 non-redundant motifs obtained from (12) as inputs to the method.

Supplementary Note 4. Claim: Everything else being equal if one pair of sequences has more shared sites for a motif m, i.e., a greater value of min (n_m^x, n_m^y)), the LLR score will be greater.

The Regulus score for the given pair of sequence *S = {x,y}* is the log likelihood ratio:

$$LLR = \log \frac{\Pr(S|M)}{\Pr(S|nB)}$$

To appreciate the claim above, first note that we may rewrite the two likelihood scores (denominator and numerator in *LLR* above) as:

$$\Pr(S|nB) = \sum_T \Pr(T|HMM)\Pr(S|T)$$
$$\Pr(S|M) = \sum_T \varphi'(\mathrm{T}) \Pr(T|HMM) \Pr(S|T)$$

where

$$\varphi'(\mathrm{T}) = \frac{\varphi(\mathrm{T})}{\sum_{T'} \varphi(T')\Pr(T'|HMM)}$$

Thus, each likelihood score can be viewed upon as an expectation of $\Pr(S|T)$ over a probability distribution, given by $\Pr(T|HMM)$ for the null model *nB* and by $\varphi'(\mathrm{T}) \Pr(T|HMM)$ for the homology model *M*. The latter distribution assigns greater probability mass to state paths T that have more shared sites between the two sequences. Thus, a pair of sequences S with greater $\Pr(S|T)$ for such state paths will receive a greater *LLR* score.

Supplementary Methods. Details of the Regulus score and its computation.

Supplementary file 1. Training CRMs and Expression sets. All 36 CRM sets in *D. melanogaster*, orthologous CRM sets in 11 other *Drosophila* species, and relevant expression data set for each CRM set.

Supplementary file 2. Source code of 3 motif-blind methods. The source code for IMM, HexMCD, and PAC-rc along with a toy example and a README file is provided.

References:

1. Kantorovitz MR, *et al.* (2009) Motif-blind, genome-wide discovery of cis-regulatory modules in Drosophila and mouse. *Dev Cell* 17(4):568-579.
2. Hooper KL, Parkhurst SM, & Ish-Horowicz D (1989) Spatial control of hairy protein expression during embryogenesis. *Development* 107(3):489-504.
3. Tomancak P, *et al.* (2007) Global analysis of patterns of gene expression during Drosophila embryogenesis. *Genome Biol* 8(7):R145.
4. Howard KR & Struhl G (1990) Decoding positional information: regulation of the pair-rule gene hairy. *Development* 110(4):1223-1231.
5. Cande J, Goltsev Y, & Levine MS (2009) Conservation of enhancer location in divergent insects. *Proc Natl Acad Sci U S A* 106(34):14414-14419.
6. Markstein M, *et al.* (2004) A regulatory code for neurogenic gene expression in the Drosophila embryo. *Development* 131(10):2387-2394.
7. Zinzen RP, Cande J, Ronshaugen M, Papatsenko D, & Levine M (2006) Evolution of the ventral midline in insect embryos. *Dev Cell* 11(6):895-902.
8. Hare EE, Peterson BK, Iyer VN, Meier R, & Eisen MB (2008) Sepsid even-skipped enhancers are functionally conserved in Drosophila despite lack of sequence conservation. *PLoS genetics* 4(6):e1000106.
9. Crocker J & Erives A (2008) A closer look at the eve stripe 2 enhancers of Drosophila and Themira. *PLoS genetics* 4(11):e1000276.
10. Hertz GZ, Hartzell GW, 3rd, & Stormo GD (1990) Identification of consensus patterns in unaligned DNA sequences known to be functionally related. *Comput Appl Biosci* 6(2):81-92.
11. Salton G & McGill MJ (1986) *Introduction to Modern Information Retrieval* (McGraw-Hill, Inc.) p 400.
12. Zhu LJ, *et al.* (2011) FlyFactorSurvey: a database of Drosophila transcription factor binding specificities determined using the bacterial one-hybrid system. *Nucleic Acids Res* 39(Database issue):D111-117.
